# Inhalation Bioaccessibility, Pollution Assessment, and Speciation of Heavy Metals in PM_2.5RD_ Road Dust from Urban and Semi-Urban Areas of Pakistan

**DOI:** 10.3390/toxics14080704

**Published:** 2026-08-10

**Authors:** Haseeb Tufail Moryani, Shu-Ming Zhu, Li-Ping Wang, Bin Xu, Changda Yu, Guang-Hui Dong

**Affiliations:** 1 Joint International Research Laboratory of Environment and Health, Ministry of Education, Guangdong Provincial Engineering Technology Research Center of Environmental Pollution and Health Risk Assessment, Department of Occupational and Environmental Health, School of Public Health, Sun Yat-sen University, Guangzhou 510080, China; tufail@mail.sysu.edu.cn; 2 Supercomputing Platform of the Center Laboratory, School of Public Health, Sun Yat-sen University, Guangzhou 510080, China; 3 School of Public Health, Sun Yat-sen University, Dongguan 523900, China; 4 Department of Public Health, Nanning Center for Disease Control and Prevention, Nanning 530023, China; 5 Institute of Space and Earth Information Science, The Chinese University of Hong Kong, Hong Kong SAR, China; 6 Joint International Research Laboratory of Environment and Health, Ministry of Education, Guangdong Provincial Engineering Technology Research Center of Environmental Pollution and Health Risk Assessment, Department of Preventive Medicine, School of Public Health, Sun Yat-sen University, 74 Zhongshan 2nd Road, Yuexiu District, Guangzhou 510080, China

**Keywords:** PM_2.5RD_, inhalation bioaccessibility, heavy metals, pollution indices, source apportionment, PHREEQC, MINTEQ

## Abstract

Ambient exposure to fine particulate matter (PM_2.5_) remains a critical global public health concern; however, the speciation and bioaccessibility mechanisms of particle-bound heavy metals within the lung environment are still insufficiently understood. In particular, the role of soluble and insoluble metal species in simulated lung fluids requires further clarification. Road dust, a major contributor to PM_2.5_, contains heavy metals that pose significant inhalation risks, leading to oxidative stress, DNA damage, and systemic toxicity. This study investigated the contamination characteristics, bioaccessibility, pollution level, and speciation of heavy metals in PM_2.5_-fractionated road dust (PM_2.5RD_) collected from Karachi (a megacity with >27 million inhabitants) and Shikarpur (a medium-sized city of ~0.3 million) in Pakistan. A grid-based sampling approach was applied across four functional zones (residential, school, hospital, and office), yielding 48 samples. Inhalation bioaccessibility was evaluated using artificial lysosomal fluid (ALF; pH 4.5, 37 °C, 24 h), revealing moderate bioaccessibility of Cr, As, and Pb. Pollution status was assessed using contamination factor (CF), integrated pollution index (IPI), pollution load index (PLI), and potential ecological risk index. Geochemical modeling with PHREEQC 3.0 and Visual MINTEQ 3.1 was employed to simulate metal speciation, release behavior, and mineral saturation states. Overall, this study provides mechanistic insights into speciation-driven toxicity of As, Pb, and Cr in simulated lung environments, offering a scientific basis for improved risk assessment and regulatory strategies for PM_2.5_-associated metal exposure.

## 1. Introduction

Road dust is a significant source of particulate matter in the atmosphere, particularly PM_2.5_ [1,2,3,4]. Particulate matter pollution has long been seen as a major concern, particularly in cities, where high traffic is responsible for the generation of various heavy metals such as Fe, Cu, Zn, Sb, and Pb through exhaust and non-exhaust emissions [5]. In addition, the existence of a large number of expired and poorly maintained cars in developing-country cities contributes to the rise in PM_2.5_ pollution levels [6]. In the last two decades, exposure to particulate matter through inhalation has become of considerable concern [7]. Research has shown PM_2.5_ pollution is most hazardous to the respiratory system as compared to other systems of the body [8,9]. Children are particularly sensitive to metal poisoning, which can negatively impact normal growth [10].

This study focuses on the pollution of heavy metals in the PM_2.5_ fraction of road dust (PM_2.5RD_), as the significance of the inhalation pathway has been well established by previous research. For this purpose, road dust samples were collected from four types of sites: hospital, office buildings, school, and residential areas. These sampling sites were selected from two cities: Karachi, the largest city in Pakistan and the second most populous city in South Asia, and Shikarpur, a smaller city in the Sindh province of Pakistan. The selected sampling sites—hospitals, office buildings, schools, and residential areas—represent key locations where populations spend considerable daily time, thereby facilitating sustained exposure to resuspended road dust particles and associated metals. These sites encompass both the general working-age population and vulnerable subgroups, including children and patients, who exhibit heightened susceptibility to the health risks of inhaled PM_2.5_-bound metals. This targeted selection enables a comprehensive assessment of differential exposure patterns across demographic groups.

According to the literature, in vitro bioaccessibility tests using simulated lung fluids (SLFs) are the most applied methodology to estimate PM_2.5_-bound metals inhalation bioaccessibility, because of being fast, simple, and cost-effective in comparison with in vivo and in vitro using cultured cells methodologies [7,11,12]. Currently, two types of SLFs, Gamble’s solution (GS) and artificial lysosomal fluid (ALF), have been widely used by researchers to estimate metal(loid)s inhalation bioaccessibility from dust or particulate matter [11,13]. Among them, ALF has been found to be more accurate in mimicking the conditions of alveolar macrophages [14,15].

This study aimed to assess in vitro inhalation bioaccessibility of heavy metals (As, Cr, and Pb) in PM_2.5RD_ from two cities of Pakistan, Karachi and Shikarpur, by using artificial lysosomal fluid (ALF). Among them, as a megacity of South Asia, Karachi is the most populous, urbanized, and industrialized metropolitan city of Pakistan. On the other hand, Shikarpur is a small city in Sindh province of Pakistan, where most of the people are associated with agriculture. Assessment of metal pollution in PM_2.5RD_ using contamination factor, integrated pollution index, pollution load index, and potential ecological risk index reveals extremely high contamination levels of Cu, Pb, Cd, Zn, and Sb across major residential, school, hospital, and office urban areas in both Karachi and Shikarpur, respectively. Furthermore, the geochemical modeling software PHREEQC and MINTEQ were used in this study to better understand the process of release mechanism and speciation of heavy metals in human lung fluid.

## 2. Material and Methods

### 2.1. Study Area

**Figure 1:**
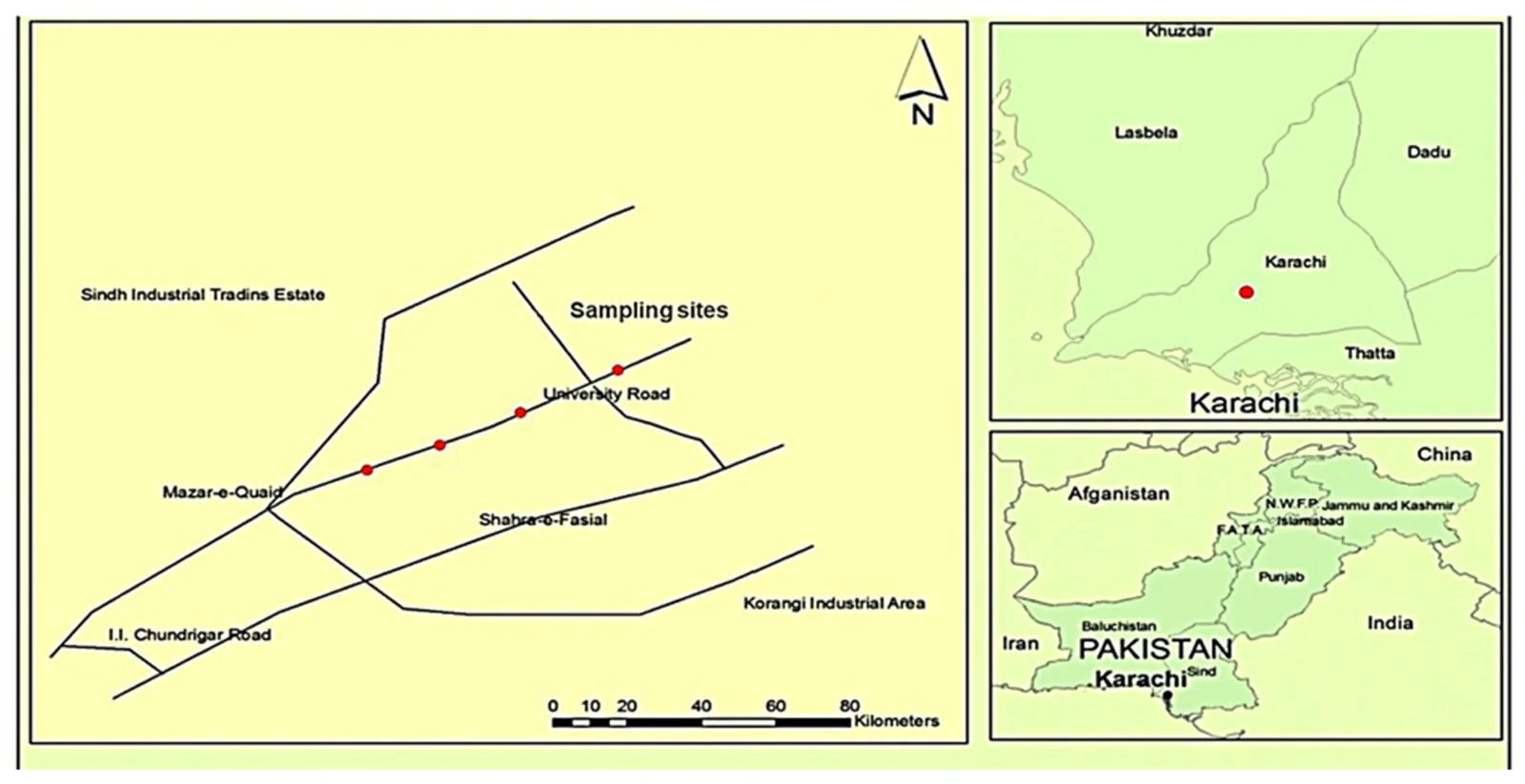
Sampling location map of Karachi [25]. The four red dots shown on the map from left to right represent the four sampling locations corresponding to residential, office, hospital, and school areas, respectively. Reproduced from [Haseeb Tufail Moryani], International Journal of Environmental Research and Public Health 2020, 17, 7124 [25].

Karachi and Shikarpur are two cities of Sindh province of Pakistan. Karachi (24°51′ N 67°02′ E) is the largest and most populous city in Pakistan (Figure 1), and the 14th largest city in the world [16]. Karachi has a population of 27 million and covers 3530 square kilometers. It has a huge road network that is more than 8000 km long [17]. The soil of Karachi is calcareous, marine in origin, and belongs to the upper tertiary period of the geological time scale [18]. Karachi’s geology comprises undulating plains, a large coastline area in the southeast, and flat or rolling plains encircled by hills in the north and west.

The Kirthar range’s hills occupy a large region of over 300 km from north to south of Karachi [6]. The city has a subtropical climate, with an average monthly temperature of 13 to 34 degrees Celsius and an average rainfall of 256 mm [19]. Recent research by K. Lurie highlights that Karachi faces some of the highest health risks from total suspended particles (TSP) [6]. More than half of Karachi’s residents live in densely packed, informal settlements, many of which sit at the heart of the city’s busiest traffic corridors [20].

Road dust and vehicle exhaust are major contributors to Karachi’s polluted air [21]. Karachi moves every day with more than 3.5 million registered vehicles on its roads [20]. Karachi’s traffic is a tapestry of vehicles, with motorbikes making up about 30%, cars 27%, public transit 13%, trucks 12%, office vans 10%, and taxis and three-wheelers 4% [22]. Even though a large share of Karachi’s traffic moves daily, about 30% of the vehicles are not friendly to the environment. Karachi’s sampling sites were picked along the city’s busiest roads, where daily traffic ranges from about 70,000 to 180,000 vehicles. On these streets, motorbikes carry the largest share of traffic, followed by cars, buses, office vans, and three-wheelers.

**Figure 2:**
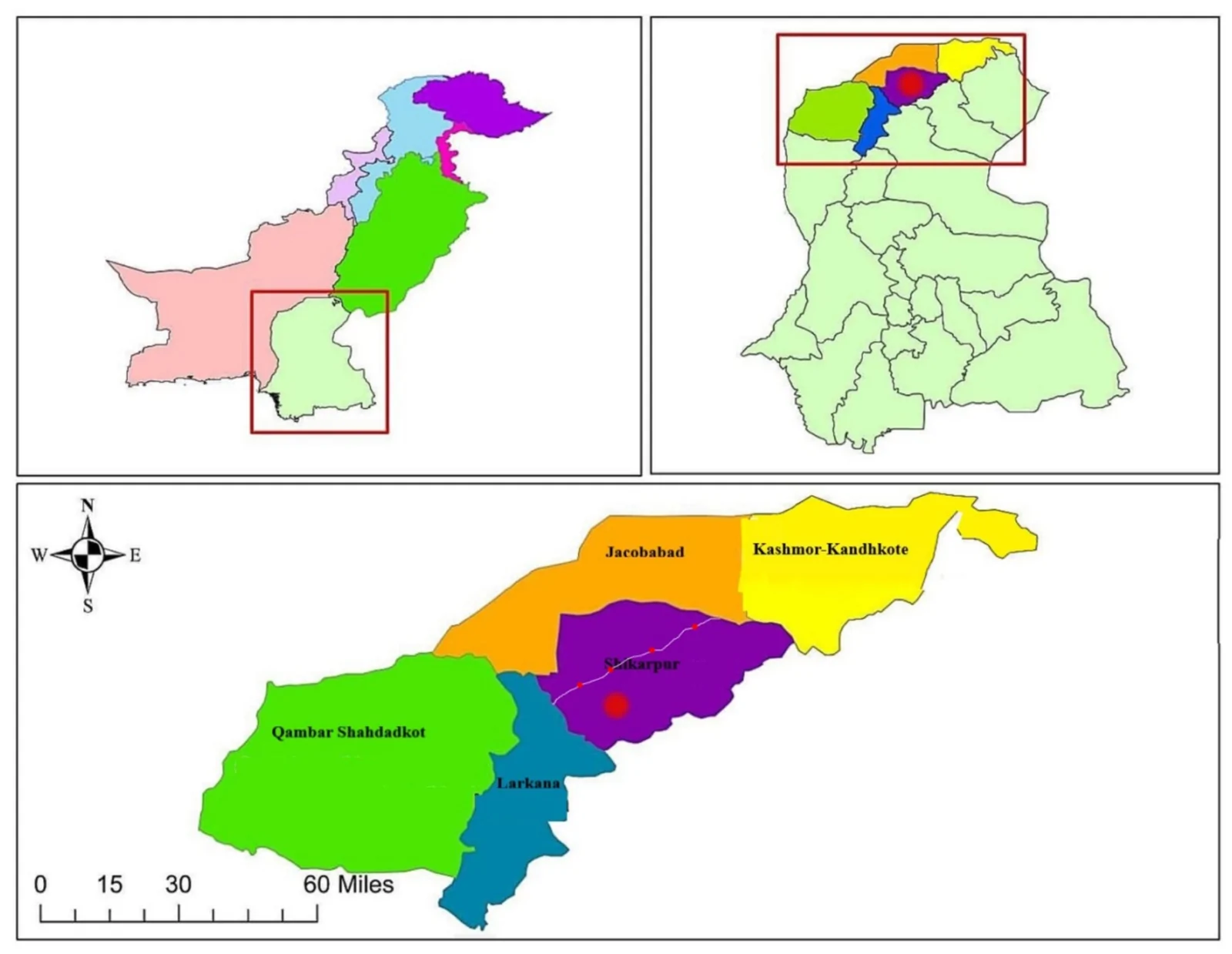
Sampling location map of Shikarpur [25]. The four red dots shown on the map from left to right represent the four sampling locations corresponding to residential, office, hospital, and school areas, respectively. Reproduced from [Haseeb Tufail Moryani], International Journal of Environmental Research and Public Health 2020, 17, 7124 [25].

Meanwhile, Shikarpur (Figure 2) is a small agricultural city (27°57′25″ N 68°38′16″ E), and the capital of Shikarpur district is situated in the northern part of Sindh province at a distance of 29 km from the bank of the Indus River. The northern part of Sindh province, including Shikarpur, mostly comprises sedimentary rocks of the Laki formation and belongs to Cretaceous and pre-Cretaceous geological periods [23]. According to the 2017 census, the population of the Shikarpur district is 1,231,481, with 207,555 households. Among them, 303,249 people are living in urban areas, and the remainder are settled in rural areas [24].

Shikarpur plays a prominent role in rice production; for this reason, a large number of people from this rural area are concerned with this work. There are no major industrial activities in the vicinity of the sampling area, and the largest sources of pollution are urban traffic on roads and in the residential areas. The sampling sites in Shikarpur were chosen from the main road of Shikarpur, named “Station Road”, where daily traffic volumes are generally between 7000 to 12,000 vehicles, with motorbikes contributing a major portion of traffic, followed by three-wheelers, cars, vans, and buses. In the current study, four spots of main roads containing residential areas, offices, hospitals, and schools along the roads with heavy traffic have been selected as research objects for road dust sampling from Karachi and Shikarpur. From each spot, two locations were selected for road dust sampling: one from the main road with heavy traffic and the second from the main entrance of the building. Figure 1 and Figure 2 illustrate the sampling locations of this study.

### 2.2. Sample Collection

Four areas in each city were selected for road dust sampling, including a school, hospital, residential area, and office buildings. In each city, sampling sites were selected from the areas with busy roads. The selection of these particular areas was done in order to cover maximum segments of our population, including adults, children, and patients. Samples were collected from urban zones with heavy traffic load. Site-specific soils that may wash onto the roadways during rainy seasons were not selected for sample collection. Two locations from each site were selected for road dust collection. One sample was taken from the nearby roads and the second sample was taken in front of the main entrance of the area’s buildings, indicated as 1 and 2 in sample identity (ID), respectively. Classification of sampling locations on the basis of major activity (such as residential, office, hospital, and school) is displayed in our previously published paper [25] (Table S1 of Supplementary Information).

All the study sites of the present research were located on the main roads in urban areas with heavy traffic. Road dust samples were collected from the hardened ground using a plastic brush and pan, a commonly used method for collecting road dust samples in the literature [26,27]. To generate a composite sample, three replicate samples were obtained from each sampling location and combined into one sample. As a result, a total of 48 road dust samples were collected from both cities’ study regions. The collected road dust samples were stored in plastic freezing bags.

The sampling was conducted in the month of February. The weather conditions were stable during the sampling period. The weather was dry with no rain events for about one week before the sampling date. Unlike sweeping, street washing is not a routine practice in either Karachi or Shikarpur. The amount of collected road dust sample from each site was >150 g. Before and in between samplings, the brush and shovel were disinfected with deionized water and a mild acidic solution. After road dust collection, all samples were taken to the laboratory and dried at room temperature. In order to separate the dust from materials, such as leaves, hair, cigarette butts, plastic particles, etc., samples were sieved through 53 mesh and stored in airtight plastic bags for analysis. All samples were stored in a cool and dry place until further analysis.

### 2.3. Preparation of PM_2.5RD_ Fractioned Road Dust

In order to obtain the PM_2.5RD_, 47 mm-sized Teflon filters were used. Before use, each 47 mm Teflon filter was dried at 150 °C for 6 h, conditioned for 48 h at 25 °C, and then blank filters were measured three times with an electronic microbalance. The road dust particles < 53 µm in size were suspended in the resuspension chamber and sampled through a PM_2.5_ size-selective cutter on filters for further analysis. After filtering the PM_2.5RD_, the weight of each filter was again measured three times with an electronic microbalance. After weighing, filters were wrapped in aluminum foil sheets and stored at −20 °C until further analysis.

### 2.4. Total Metal Concentration

Total metal concentration was determined using inductively coupled plasma mass spectroscopy (ICP-MS, NexION-350X series, PerkinElmer, Waltham, MA, USA) following aqua-regia digestion (MARS-6 microwave (CEM) and USEPA method 3051 (USEPA, 1998)). Briefly, Teflon filters containing the PM_2.5RD_ samples were divided into two equal halves; each half was digested using 5 mL of aqua regia solution. After the digestion process, samples were diluted in a 2% nitric acid HNO_3_ solution for analysis with ICP-MS. The minimum, maximum, mean, and standard deviation (SD) of total metal concentrations associated with PM_2.5RD_ for Karachi and Shikarpur are presented in Table 1 and Table 2, respectively.

### 2.5. Quality Control

Quality assurance and quality control were monitored using calibration blanks, laboratory reagent blanks, calibration standards, and internal standards. All samples were examined in triplicate, including filter blanks and standard reference material (SRM). Trace-grade acids (hydrochloric and nitric acid) were used for digestion and analysis. The standard reference material (GBW07451) was purchased from the National Center of Standard Materials of China (NCSMC) Beijing, China [25]. The analysis was performed in duplicate in order to confirm the accuracy of the aqua-regia digestion method and spiked samples. The % recovery was calculated using concentrations of heavy metals in SRM, spiked samples, and samples with the same procedure as an earlier study [15]. Recoveries from internal standards and SRM ranged from 91.0% to 100.4% and 90% to 110%, respectively.

### 2.6. In Vitro Bioaccessibility Test

The in vitro inhalation bioaccessibility test was conducted on PM_2.5RD_ samples by using ALF. The selection of ALF as a simulated lung fluid (SLF) was chosen on the basis of its effective results towards assessing the bioaccessibility of heavy metals in PM_2.5RD_. ALF solution simulates intercellular conditions in lung cells occurring in conjunction with phagocytosis, thereby representing relatively harsh conditions. Artificial lysosomal fluid (ALF) [28] was freshly prepared by dissolving the ingredients listed in Table S2 in one liter of ultrapure (Up) water and adjusting the pH of the solution to 4.5. Previous research has established by systematically testing S/L solid (g) to liquid (mL) ratios from 1:100 to 1:5000 using ALF and found that metal(loid) solubility generally plateaued at 24 h when a 1:5000 ratio and end-over-end rotation were used, supporting this as a conservative and suitable ratio for PM_2.5_ inhalation bioaccessibility [15]. The prepared solution was kept in an incubator at 37 °C overnight to reach temperature. The method of agitation and the bioaccessibility testing time widely vary in the literature. In the present study, an orbital shaker at a speed of 45 rpm and a testing time of inhalation bioaccessibility was selected as 24 h as suggested by an earlier study [15].

The in vitro inhalation bioaccessibility test was conducted in triplicate, and procedural blanks were included in duplicate. Briefly, the second half of each Teflon filter was placed in a 50 mL centrifuge tube, and 40 mL of ALF solution was added into each centrifuge tube using an automatic pipette. The tubes were sealed using parafilm tape and fixed inside the incubator in the dark. After 24 h, all the tubes were collected from the incubator and centrifuged at a speed of 10,000 rpm for 10 min in order to separate solids from liquids. The supernatant was filtered using 10 mL syringes and 0.45 µm filters, and it was diluted with 0.1 M HNO_3_ solution. The diluted samples were stored at 4 °C in a refrigerator until further analysis, using ICP-MS (NexION-350X series).

### 2.7. Bioaccessible Fraction

The inhalation bioaccessibility percentage (%) was calculated by dividing the bioaccessible concentration of heavy metal in ALF by the total heavy metal concentration determined by the aqua-regia digestion procedure, as described in Equation (1).

(1) $$Inhalation\;bioaccessibility=\frac{In\;vitro\;metal\;[mg/kg]}{Total\;metal\;\left[\frac{mg}{kg}\right]\;in\;PM2.5RD}$$

### 2.8. Pollution Characteristics

Many techniques of quantification have been used to measure the pollution rate of heavy metals in natural environmental samples, such as dust, soils, and sediments. In this study, the contamination factor (CF), integrated pollution (IPI), pollution load index (PLI), and potential ecological risk index were employed to assess the degree of PTMs pollution in PM_2.5RD_ of Karachi and Shikarpur. Since dust contains tiny soil particles, it is possible that it can be transferred to various environmental types, such as residential areas, schools, offices, and hospitals, and accumulate on bare surfaces; soil background values can be used for dust. As environmental baseline data are yet to be established, some studies based on Pakistan have used the elemental values from [29,30].

#### 2.8.1. Contamination Factor (CF)

The contamination factor, also known as the pollution index, refers to the ratio of the elemental concentration of the sample to the background value of the element, and the formula for calculation is described in Equation (2):

(2) $$CF=\frac{C_{n}}{B_{n}}$$

where C_n_ is the sample concentration, and B_n_ is the background value of the toxic metal. The classification of contamination factor (CF) is also given in Table S3.

#### 2.8.2. Integrated Pollution Index (IPI)

The arithmetic mean of the contamination factor (pollution index) for every individual toxic metal is defined as the integrated pollution index, and the classification is given in Table S4. The formula we used to calculate the integrated pollution index is given below in Equation (3):

(3) $$IPI=\frac{\sum PIi}{n}$$

where PI_i_ represents the pollution index of i element, and n is the total number of values (samples).

#### 2.8.3. Pollution Load Index (PLI)

The pollution load index describes the overall contamination level and cumulative explanation of the pollution status of heavy metals. It was proposed by [31] and classified as either pollution or no pollution of toxic heavy metals. It can be calculated by IPI values, as mentioned in Equation (4):

(4) PLI=IPIE1×IPIE2×IPIE3×IPIE4×⋯⋯×IPIEn1n

where IPI En is the mean value of pollution index/contamination factor of PTMs, and n is the number of toxic heavy metals [32].

#### 2.8.4. Ecological Risk Index (Er)

The ecological risk of HMs in road dust was assessed by calculating the potential ecological risk factor of individual metal (Er). The ecological risk of heavy metals was computed by using Equations (5) and (6):

(5) $$RI={\sum Er}^{i}$$

(6) $${Er}^{i}={Tr}^{i}\times Cf$$

where Tr is the metal toxic factor. The Tr for HMs follow the order of Zn = 1 < Cr = 2 < Cu = Ni = Pb = 5 < Cd = 30. Cf is the contamination factor of the heavy metals. In this study, the minimum, mean, and maximum Er and RI were calculated for each heavy metal. Table S5 displays the grades for ecological risk factor of individual metal whereas Table S6 provides the grading standards of potential ecological risk from HMs.

### 2.9. Geochemical Speciation Modelling

#### 2.9.1. PHREEQC Modeling

PHREEQC version 3.4.0 with the database minteq.v4.dat was employed in geochemical modeling. The geochemical modelling inputs for PHREEQC and Visual MINTEQ were based on the experimentally measured total concentrations of As, Pb, and Cr obtained by ICP-MS from the PM_2.5RD_ samples, together with the ALF composition, pH (4.5), temperature (37 °C), and other solution conditions used in the in vitro bioaccessibility assay. An excess of the heavy metals in one liter of the ALF in oxidized conditions at 37 °C was considered. For simulations with site mineralogy, concentrations of heavy metals were selected based on inductively coupled plasma mass spectrometry (ICP-MS) analysis.

The ALF input file was used the same as in the previous study [33]. Moreover, mineral saturation indices (SI) were computed using the equilibrium geochemical speciation modeling PHREEQC and MINTEQ database. SI is the ratio of the ion activity product’s logarithm to the solubility product’s logarithm. One of the most commonly used markers in hydrogeochemical studies is saturation index (SI). It investigates the condition of the saturation of heavy metals in aqueous solutions. SI = lgIAP lgK_sp_ for minerals in aqueous solution, where IAP is the activity product of related ions in the mineral dissolution solution, and K_sp_ is the equilibrium constant of the metal dissolution process at a given temperature. The metal is in equilibrium in the aqueous solution when SI = 0. When the SI is less than zero, the metal is not saturated in the aqueous solution and will dissolve; when the SI is more than zero, the metal is supersaturated in the aqueous solution and will precipitate.

#### 2.9.2. Simulation of Metal(loid)s Solubility by Visual MINTEQ

In the current study, visual MINTEQ 3.1 (https://vminteq.com/, accessed on 1 June 2026) was used to simulate the contact between leaching agents and road dust fraction in order to understand how the solubility of heavy metals in the leaching solution changes depending on the composition of simulated lung fluid and the speciation of heavy metals. The input files for ALF and Gamble’s solution were used the same as in the previous study [33]. The pH was set at 7.4 and 4.5 for all runs of Gamble’s solution and ALF, respectively, and temperatures were set. The metal(loid)s speciation in samples was unknown; therefore, different metal(loid) species were used to simulate the leaching process. The solubility fraction of metal(loid)s and species distribution were obtained in each simulation. The speciation of three important heavy metals, As, Pb, and Cr, was calculated using Visual MINTEQ 3.1. In addition to this, the saturation index (SI) at different pH values was computed by Visual MINTEQ 3.1 to predict the possible solid phase of heavy metals.

(7) $$SI=log\left(IAP|Ksp\right)$$

The saturation index is classified with the following criteria: when the ion activity product (IAP) > K_sp_ (solubility product), SI > 0, the system is supersaturated. The larger the SI, the greater the tendency towards mineral phase precipitation. 

In this study, the simultaneous use of PHREEQC and MINTEQ software for geochemical modeling of artificial lysosomal fluid (ALF) and Gamble’s solution was employed in order to leverage their complementary strengths in simulating complex aqueous chemical systems. PHREEQC is widely recognized for its versatility in modeling diverse geochemical processes, including aqueous speciation, solubility, and reaction kinetics. In contrast, MINTEQ demonstrates particular proficiency in metal speciation and equilibrium modeling within environmental and biological contexts. The dual-software approach enables a more comprehensive understanding of the chemical behavior of the studied systems, facilitates cross-validation of modeling outcomes, and enhances the reliability of results, as each program provides distinct databases and computational algorithms optimized for specific geochemical conditions.

## 3. Results and Discussion

### 3.1. Bioaccessibility of Potential Toxic Elements

**Figure 3:**
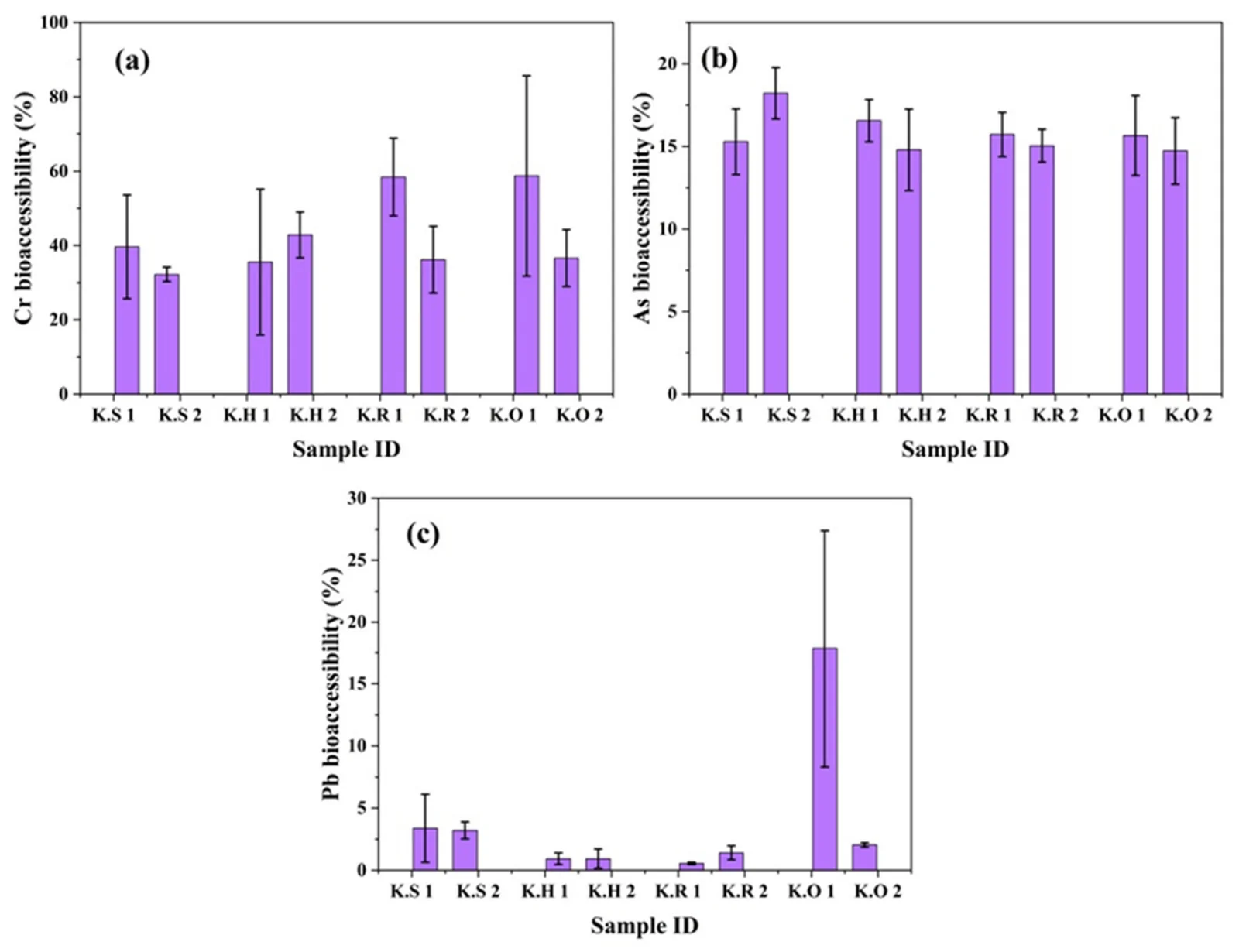
Inhalation bioaccessibility (%) charts for Karachi: (**a**) chromium; (**b**) arsenic; (**c**) lead.

**Figure 4:**
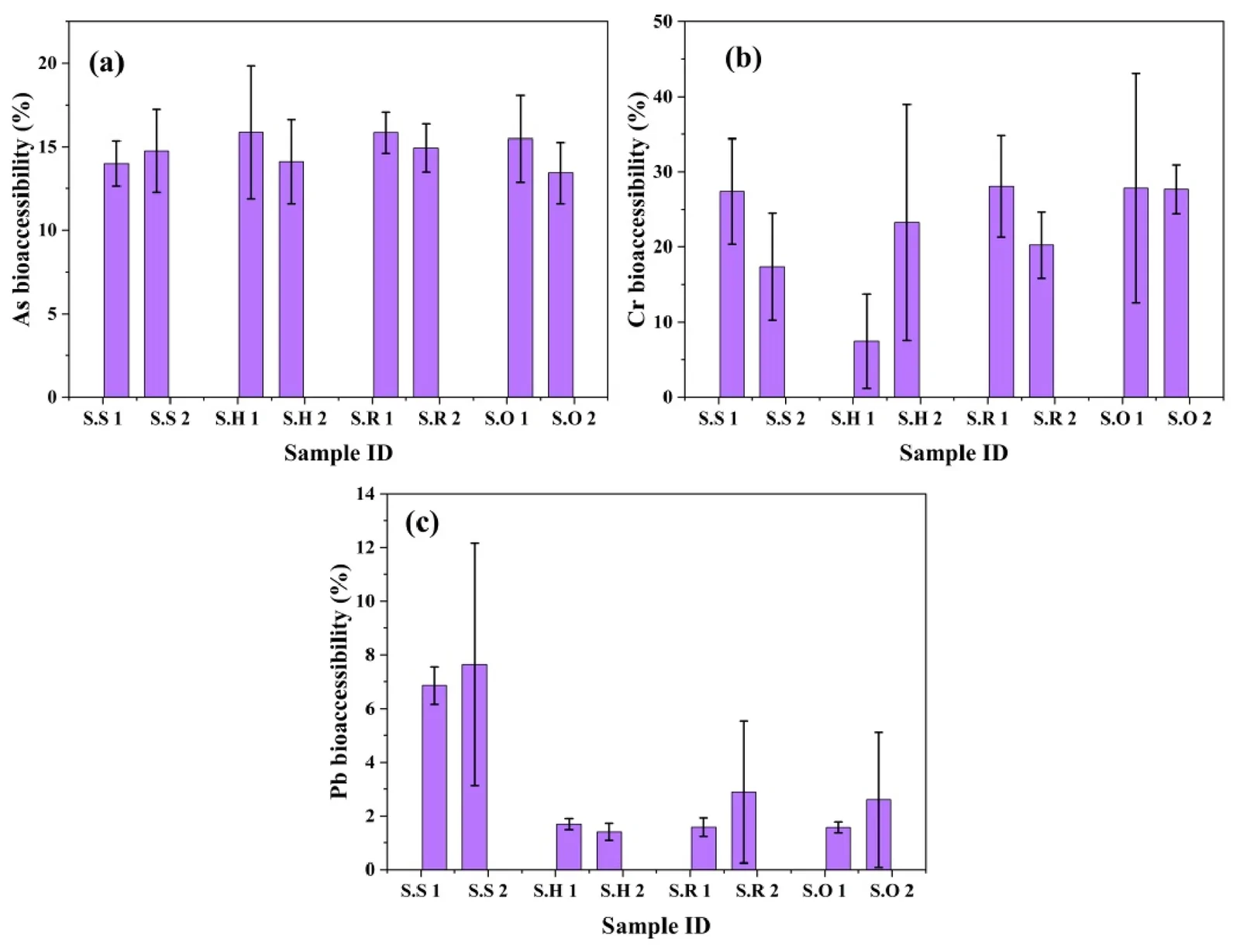
Inhalation bioaccessibility (%) charts for Shikarpur: (**a**) chromium; (**b**) arsenic; (**c**) lead.

The bioaccessibility of heavy metals associated with PM_2.5RD_ of Karachi and Shikarpur in artificial lysosomal fluid (ALF) is displayed in Figure 3 and Figure 4, respectively. In Table 3, the bioaccessibility of heavy metals associated with PM_2.5RD_ from this study is compared to that of numerous cities throughout the world. At the studied sites of Karachi and Shikarpur, the average heavy metal bioaccessibility (%) in artificial lysosomal fluid (ALF) solution followed the same order: Cr > As > Pb. An inhalation bioaccessibility test using ALF was conducted in order to measure the bioaccessible concentrations of potential toxic elements (PTEs), such as As, Cu, Cd, Cr, Pb, Sb, and Zn. Measured concentrations in artificial lysosomal fluid (ALF) were below detection limits for most elements except As, Cr, and Pb.

The difference between the bioaccessible concentrations of Pb in samples from all four areas of both cities (Karachi and Shikarpur) is much lower than the total concentrations (Figure 3 and Figure 4). Consistent with our findings, a previous study on the bioaccessibility of Pb in PM_2.5_ has also reported the same behavior of Pb during the inhalation bioaccessibility test [34]. Moreover, for samples from all four areas of Karachi, larger variations exist in concentrations of total Pb than bioaccessible Pb. The values of the bioaccessible Pb concentrations in the four sampling areas of Karachi (Figure 3) suggest that while the total Pb concentrations are very different in the sampling areas, the concentrations of the bioaccessible Pb fraction are similar, meaning that while the total Pb concentrations in the PM will easily be influenced by contamination, very high proportions of the accumulated Pb are inert in the ALF.

The bioaccessibility of As ranged from 14.72% to 18.22% in the study areas of Karachi and 13.43% to 15.86% in the study areas of Shikarpur. In research conducted in the city of Kank, similar findings were reported for As bioaccessibility, with values ranging from 13 to 18% (Table 2), indicating that these sites all sit in a low-to-moderate inhalation bioaccessibility band where only a minority of total As is soluble in ALF. The low extraction rate of As could be due to the extraction process, which may perform best in ions rather than anions, according to an earlier published analysis [35]. According to the findings of a previous study, the fluid present in the human lung has the ability to reduce arsenic [36], so besides other causes, this should also be considered a prominent reason for low bioaccessibility of arsenic. The bioaccessibility of As varies slightly among the study areas of both cities, which indicates the same source of origin. Wiseman and Zereini [37,38] obtained similar results; they found elevated solubilities for As in ALF solution.

Pb bioaccessibility ranged from 3.20% to 17.84% in the study areas of Karachi and 1.40% to 7.40% in the study areas of Shikarpur. The results of a recent research conducted in Shanghai are closely connected to the values of Pb bioaccessibility observed in Karachi (Table 3). According to a recently published study, Pb has low bioaccessibility rates in soil or dust [39]. The samples showed significantly higher Pb bioaccessibility in the office area of Karachi than in school, hospital, and residential areas. Whereas, in the case of Shikarpur, a higher amount of Pb was measured in the samples of the school area as compared to hospital, offices, and residential areas. The minute difference in bioaccessible Pb among the sampling sites of Karachi, compared with the big differences in total Pb, suggests that added contamination is mainly stored in relatively inert matrices such as Fe/Mn oxides, silicates, or tightly bound carbonate phases that only partially dissolve in ALF.

The bioaccessibility of Cr ranged from 32.20% to 58.43% in the study areas of Karachi and 7.46% to 28.04% in the study areas of Shikarpur. These findings are in line with those of a recent research (Table 3), which indicated that inhalation bioaccessibility of Cr was greater in urban areas than in rural areas [40]. The bioaccessibility of Cr showed high variance among the areas of both cities. The samples showed significantly higher Cr bioaccessibility in the office and residential areas of Karachi. However, in the case of Shikarpur, a higher amount of Pb was measured in the samples of residential and school areas compared to hospital and office areas.

Karachi’s elevated and variable Cr, peaking in office and residential areas, reflects industrial and traffic emissions, particularly brake wear, delivering soluble Cr(VI) to fine PM_2.5RD_; unlike Shikarpur, where crustal resuspension and dilute sources yield inert Cr(III). High Cr bioaccessibility in Karachi’s office and residential areas likely stems from Cr hosted in labile chromate (Cr(VI)) forms or poorly crystalline Fe/Mn oxides that dissolve readily in acidic ALF, contrasting with the refractory chromite (Cr(III)) spinels dominant in Shikarpur’s lower-solubility samples. This interpretation remains hypothetical because chromium speciation and mineralogical associations were not directly measured. Therefore, any reference to Cr(VI), Cr(III), amorphous oxides, or chromite should be regarded as a literature-based explanation supported by geochemical modelling rather than an experimentally proven result.

This study investigated the dissolution of inhalable heavy metals from a variety of PM_2.5RD_, employing artificial lysosomal fluid (ALF), which is a prominent simulated lung fluid (SLF) that simulates the lysosomal environment inside human lungs. Although ALF provides a conservative estimate of inhalation bioaccessibility, the exclusive use of this acidic simulated lung fluid is a limitation, because a neutral medium such as Gamble’s solution may yield different dissolution patterns, especially for pH-sensitive heavy metals. The dissolution of inhalable heavy metals in ALF suggests that a fraction of these heavy metals may become bioaccessible in the lung environment and could therefore have the potential for systemic uptake, although this study did not directly assess biological transport or toxicological effects.

**Table 3:** ALF bioaccessibility (%) of studied toxic heavy metals in PM_2.5RD_ compared to other cities.

City/Country		Elements		Fraction Size
	As	Pb	Cr	
Kank [41]	5.9	0.5	-	PM_2.5_
Beijing [42]	36	70	48	PM_2.5_
Harbin [40]	29.29	31.87	214.28	PM_2.5_
Cantabria [11]	-	87.5	-	PM_2.5_
Xiamen [43]	-	79.16	70.87	PM_2.5_
Jiyuan [44]	-	0.46	-	PM_2.5_
Shanghai [45]	-	20.4	-	PM_2.5_
France [46]	38.8	95.5	-	PM_2.5_
Karachi (This study)	15.7	3.7	42.5	PM_2.5_
Shikarpur (This study)	14.8	3.2	22.4	PM_2.5_

### 3.2. CF, IPI, and PLI

**Figure 5:**
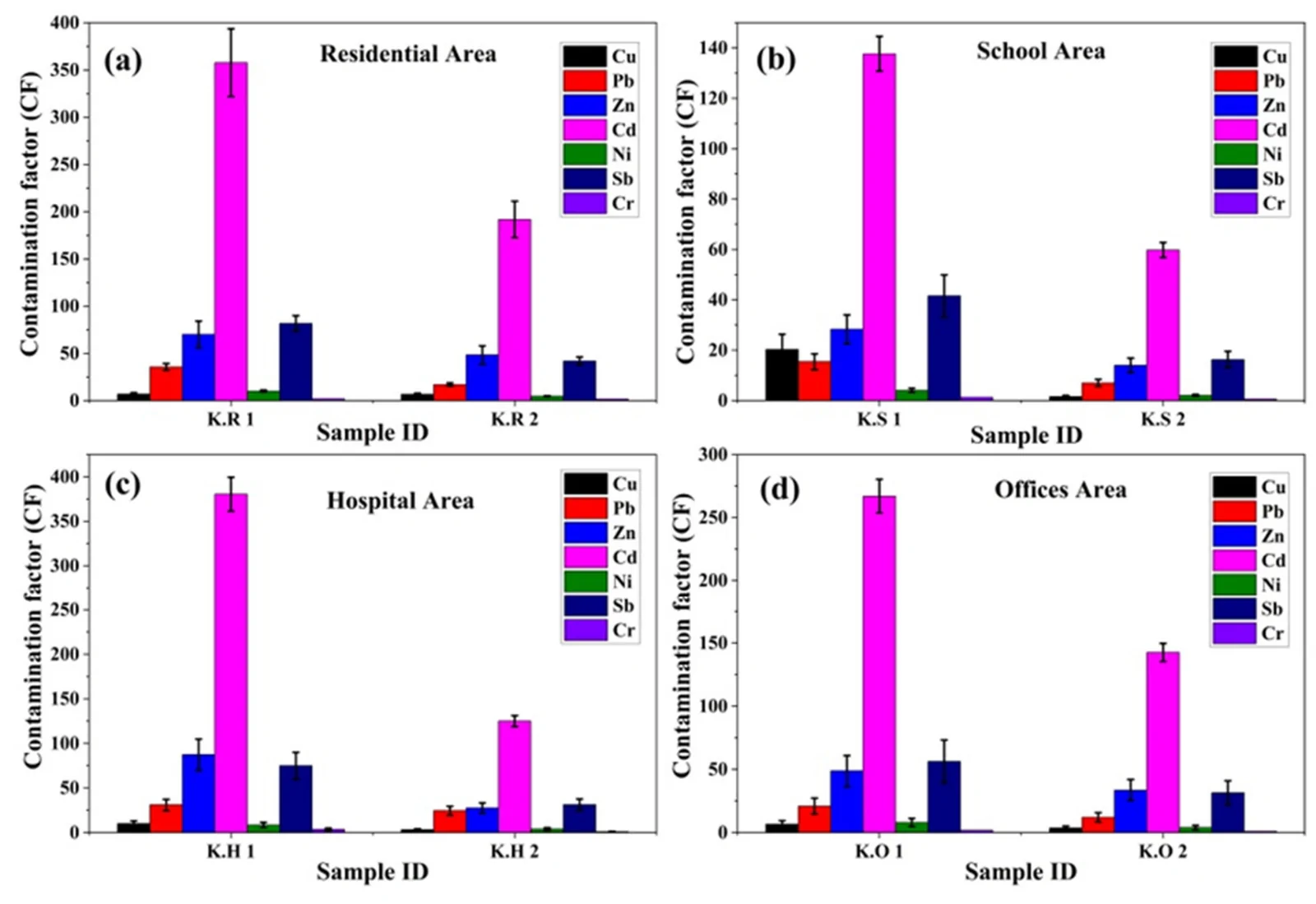
Contamination factor (CF) of heavy metals in PM2.5RD fractioned road dust from four areas of Karachi: (**a**) residential area, (**b**) school area, (**c**) hospital area, (**d**) office area.

**Figure 6:**
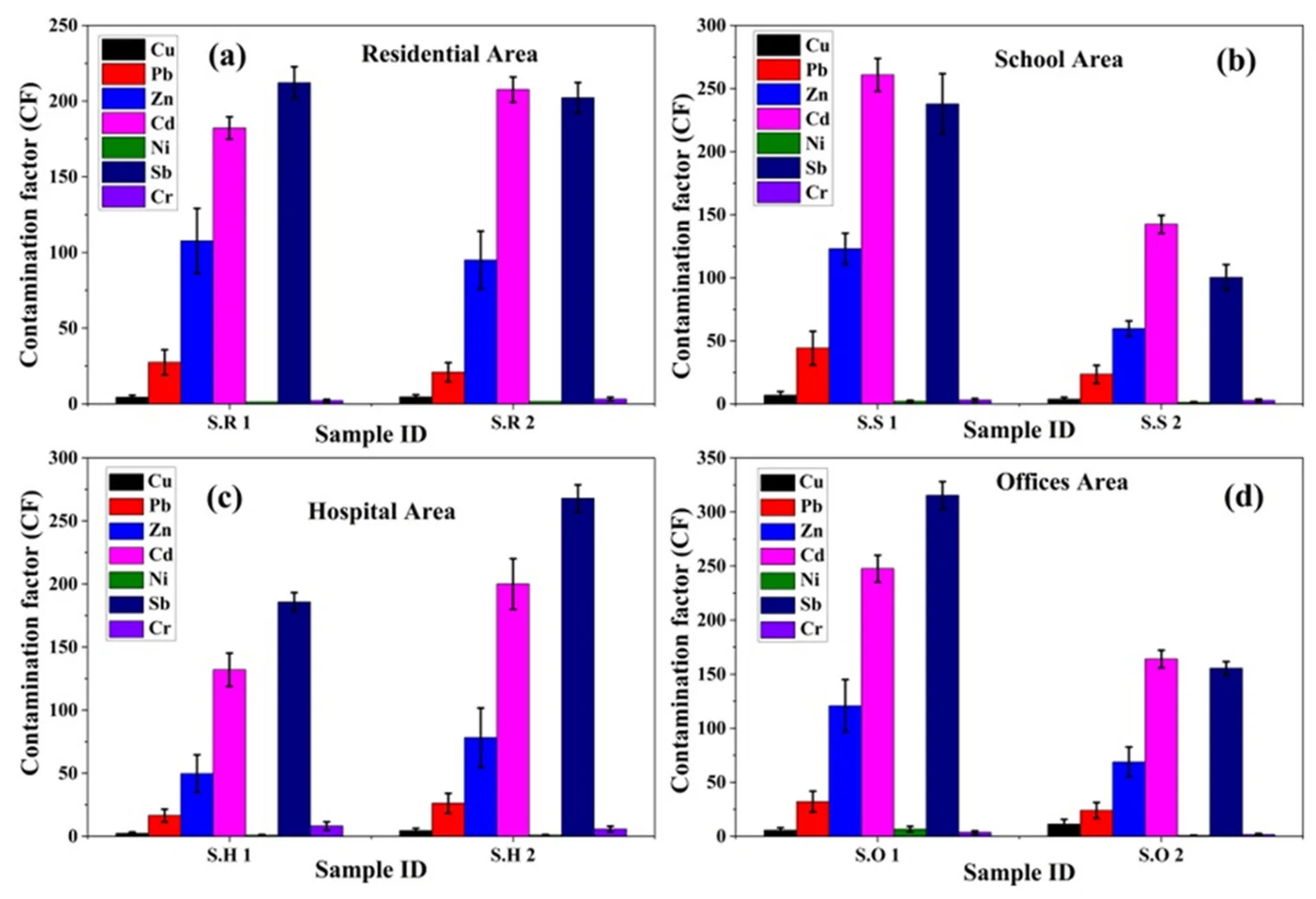
Contamination factor (CF) of heavy metals in PM2.5RD fractioned road dust from four areas of Shikarpur: (**a**) residential area, (**b**) school area, (**c**) hospital area, (**d**) office area.

**Figure 7:**
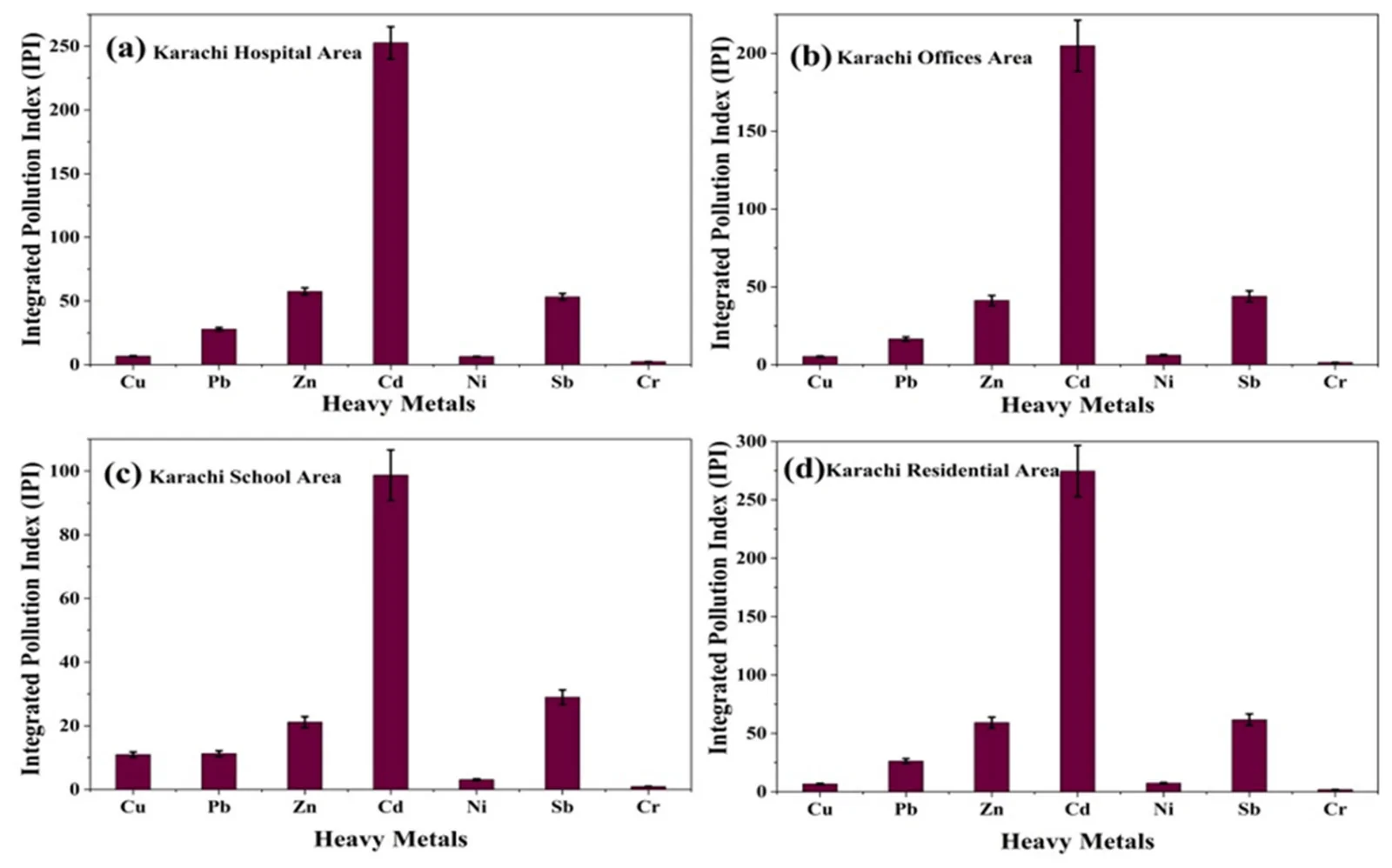
Integrated pollution index (IPI) of heavy metals in PM_2.5RD_ fractioned road dust from four areas of Karachi: (**a**) hospital area, (**b**) office area, (**c**) school area, and (**d**) residential area.

**Figure 8:**
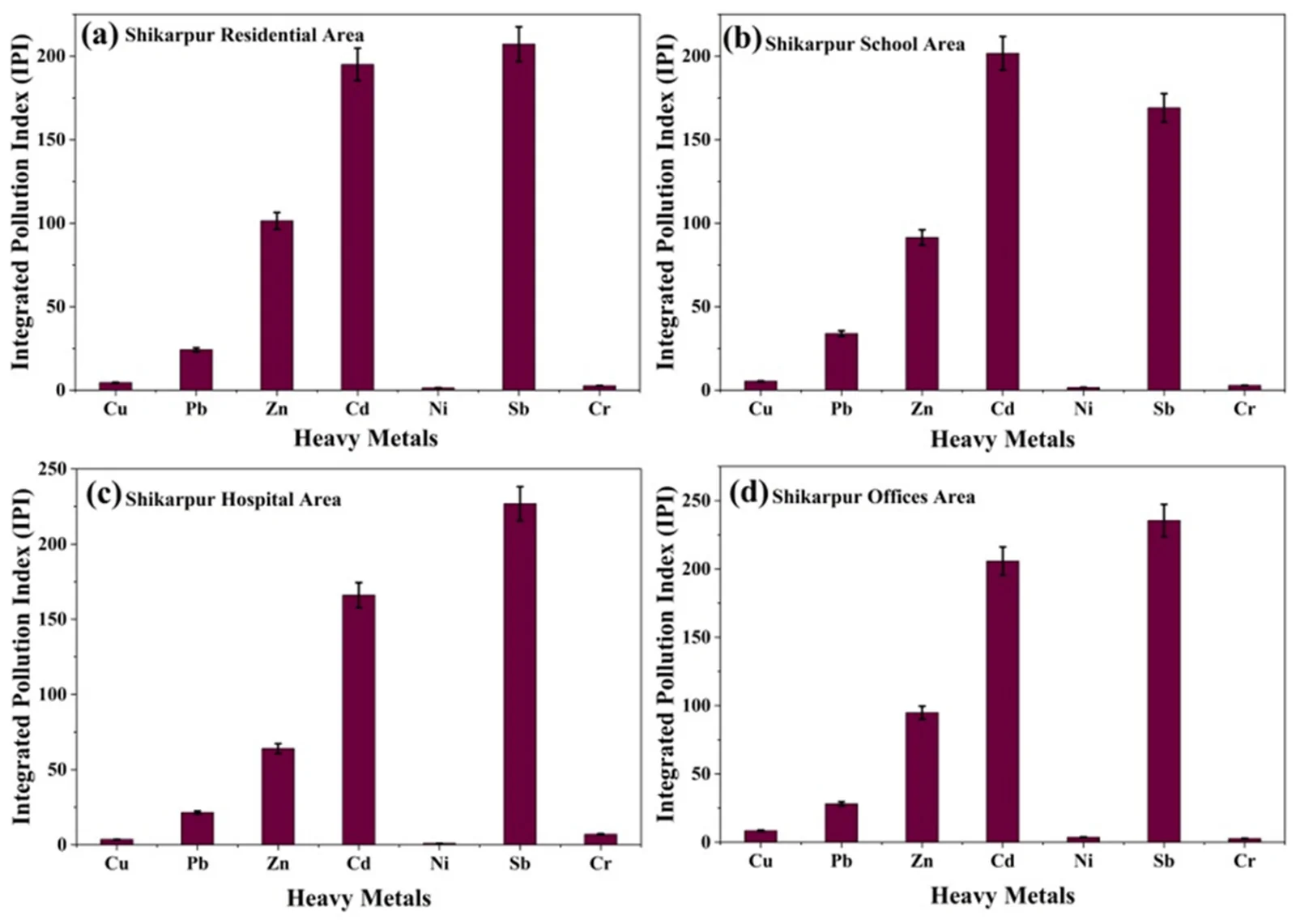
Integrated pollution index (IPI) of heavy metals in PM2.5RD fractioned road dust from four areas of Shikarpur: (**a**) residential area, (**b**) school area, (**c**) hospital area, (**d**) office area.

The CF, IPI, and PLI value ranges for explanations of the degree of pollution are provided in Table S2 and Table S3 in Supplementary Information, respectively. Figure 5 and Figure 6 show the contamination factor (CF) of PM_2.5RD_ samples in accordance with the various heavy metals and land-use types of both cities, Karachi and Shikarpur. The results revealed that there was low to high pollution found for Cu, Pb, Zn, Cd, Ni, Sb, and Cr, as their values of contamination factor were found between 0 and >3. Cu was highly polluted in all four areas (residential, school, hospital, and office) of both cities, and its IPI values exceeded 2 in all studied areas, indicating a high degree of copper contamination. In this study, the value of 2 was used as the classification boundary for IPI, while the plotted IPI values in Figure 7 and Figure 8 represent the actual calculated index and may exceed 2 considerably in heavily contaminated samples, reflecting the cumulative enrichment of multiple metals rather than a capped scale.

The contamination factor (CF) and pollution load index (PLI) provide complementary insights into the pollution status of PM_2.5RD_ in the two cities. The CF values indicated that several heavy metals, particularly Cu, Pb, Cd, Zn, and Sb, were substantially enriched relative to background levels across residential, school, hospital, and office sites. This pattern points to a persistent anthropogenic contribution, particularly by traffic-related emissions with non-exhaust sources, such as brake and tire wear. The elevated CF values in everyday urban microenvironments indicate that the contamination is not restricted to industrial zones but is pervasive within locations where people reside, work, study, and seek medical care. The PLI further revealed that the cumulative contamination burden across all studied heavy metals was considerable, reinforcing the conclusion that the dust matrix functions as a multi-element reservoir of potential exposure. Collectively, CF and PLI highlight both the intensity of individual heavy metal enrichment and the cumulative pollution pressure, emphasizing the urgency of targeted mitigation strategies in traffic-influenced urban areas, especially around sensitive sites such as schools and hospitals.

The pollution indices collectively indicate that PM_2.5RD_ in both cities was highly impacted by anthropogenic contamination, with the heavy pollution load consistently occurring in traffic-intense urban environments. The consistently elevated values across residential, school, hospital, and office sites suggest that exposure is not limited to industrial hotspots but is embedded within everyday urban settings where people live, study, work, and seek medical care. This is particularly important from a public health perspective, because the sampled environments represent locations where vulnerable groups, including children, patients, and commuters, spend prolonged periods of time and may repeatedly inhale resuspended PM_2.5_-bound metals. The index patterns therefore indicate not only contamination of dust, but also the presence of a persistent exposure reservoir capable of re-entering the breathing zone under routine human activity and wind disturbance.

**Figure 9:**
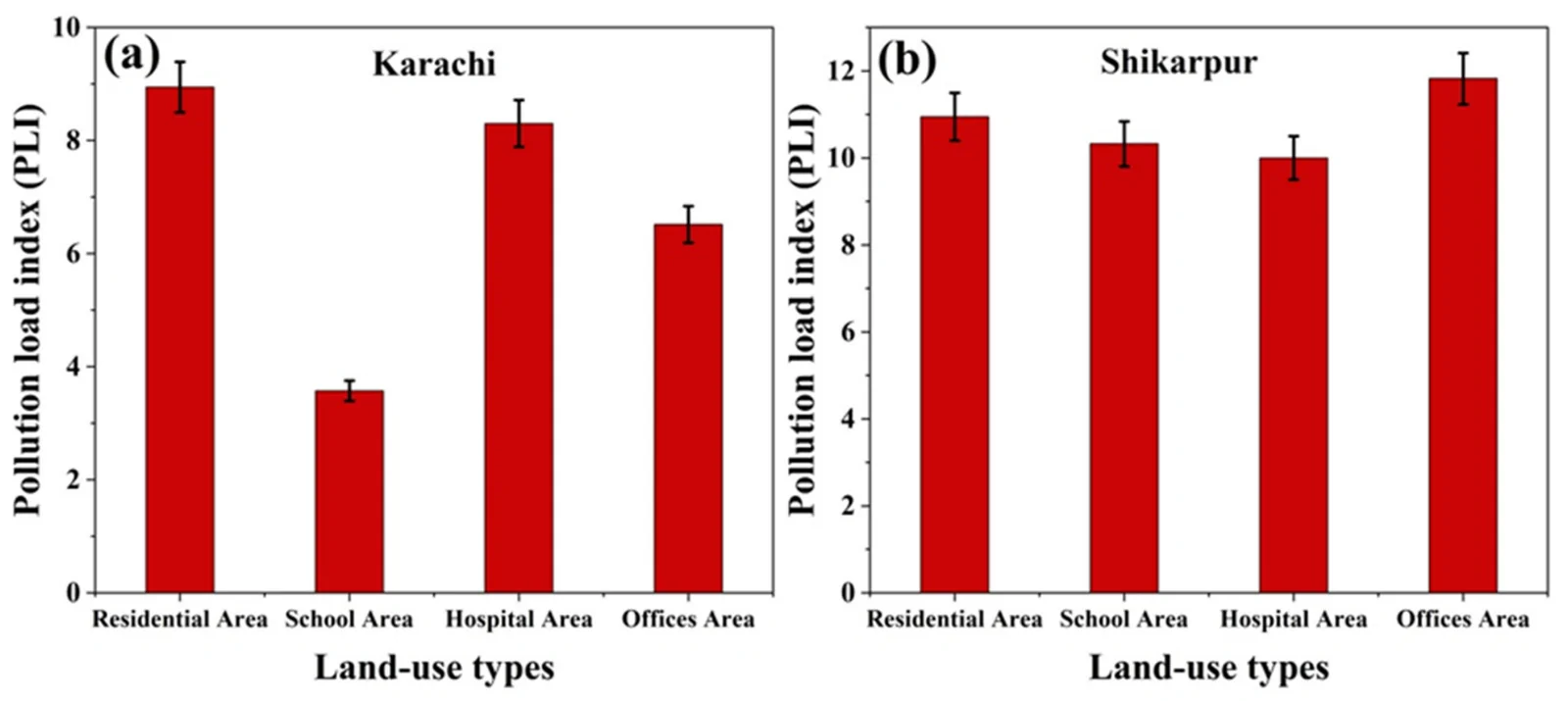
Pollution load index (PLI) of heavy metals in PM_2.5RD_ fractioned road dust from four areas of Karachi and Shikarpur: (**a**) Karachi, (**b**) Shikarpur.

The combined evidence from the indices shows that several heavy metals were present at levels exceeding background expectations to a degree that is environmentally meaningful, reflecting ongoing input rather than isolated legacy deposition. In practical terms, the high contamination burden implies that urban dust management should be considered an air quality and health protection issue rather than only a surface cleanliness issue. The results also suggest that mitigation strategies should prioritize traffic corridors, school surroundings, hospital perimeters, and other pedestrian-dense locations where dust accumulation and resuspension are likely to be frequent. The cumulative pollution load reflected in PLI (Figure 9) further demonstrates that the overall contamination status cannot be understood by examining single metals in isolation, because the dust matrix contains a mixture of toxic elements that may jointly contribute to adverse effects.

### 3.3. Potential Ecological Risk Assessment

To gain a better understanding of Karachi and Shikarpur PM_2.5RD_ pollution and related risks, Table 4 and Table 5 present ecological risks of Karachi and Shikarpur PM_2.5RD_ based on Hakanson’s approach. Table S5 and Table S6 in Supplementary Information illustrate both the E_r_ of the individual metal and (RI), respectively. The mean E_r_ values of heavy metals for Karachi and Shikarpur decrease in the following order: Cd > Pb > Zn > Cu > Ni > Cr and Cd > Pb > Zn > Cu > Cr > Ni, respectively. Cd had a considerably high potential risk in the study areas of both cities. A similar type of result for Cd has been reported by a recent, which also measured heavy enrichment of Cd as compared to other heavy metals [47].

The present study also endorses the results of a previous study, which also found high E_r_ and RI values of Cd as compared to other heavy metals [48]. The E_r_ values of Cu in the study areas of Karachi were in the range of considerable potential ecological risk; whereas in the study areas of Shikarpur, Cu showed moderate potential ecological risk. The E_r_ values of Pb in the study areas of Karachi were found between the ranges of low potential ecological risk and high potential ecological risk; whereas in the study areas of Shikarpur, E_r_ values of Pb showed considerable potential ecological risk to high potential ecological risk. Consistent with the findings of our study at Shikarpur, recent research has also determined considerable potential ecological risk for Pb [47].

The E_r_ values of Ni in the investigated areas of Karachi and Shikarpur were estimated at low potential ecological risk. The E_r_ values of Cd in the study areas of Karachi and Shikarpur were measured in the range of significantly high potential ecological risk. The E_r_ values of Cr in the investigated areas of Karachi and Shikarpur were determined at low potential ecological risk. The E_r_ values of Zn in the investigated areas of Karachi were between the ranges of low potential ecological risk and considerable potential ecological risk; whereas in the study areas of Shikarpur, E_r_ values of Zn were found between the ranges of moderate potential ecological risk and considerable potential ecological risk. The RI values of all heavy metals studied in the present study showed very high potential ecological risk; these results are consistent with the findings of a previous study, which has also determined very high potential ecological risk for all these elements [48].

### 3.4. Modelling of Geochemical Speciation

The results of geochemical modeling for arsenic (As), lead (Pb), and chromium (Cr) are presented in Table 6 and Table 7. The geochemical modelling results complement the experimental bioaccessibility data by providing mechanistic insight into the likely aqueous speciation and saturation behavior of As, Pb, and Cr under the same ALF conditions, thereby helping to explain the observed differences in metal release. The speciation distribution for samples collected from Karachi and Shikarpur is provided in Tables S7 and S8 of the Supplementary Material, respectively. According to the PHREEQC calculations, all As in the PM_2.5RD_ samples occurred as As(III) and As(V). The heavy metals dissolution model suggested that the highest amount of arsenite As(III) among the samples from the study areas of Karachi in ALF was soluble in the sample of the residential area of Karachi, similarly, the highest amount of arsenate As(V) in ALF was also found in the residential area of Karachi. Meanwhile, for the areas of Shikarpur, the highest amounts of As(III) and As(V) were also found in the residential area of Shikarpur.

The study of aqueous As speciation showed that the predominant species in ALF for the samples of the study areas of Karachi and Shikarpur were H_3_AsO_3_, H_2_AsO_3_^−^, H_4_AsO_3_^+^, and HAsO_3_^2−^ (Tables S7 and S8), which may indicate the mobilization of As if these species are stable under the pH conditions of the experiment. Furthermore, under the given experimental conditions, PHREEQC calculations showed that H_3_AsO_3_ and H_2_AsO_4_^−^ were the most stable species of As under low pH conditions of ALF. Moreover, PHREEQC modeling results under the experimental conditions of ALF showed that the most abundant species from the As(III) species in the samples of Karachi and Shikarpur was H_3_AsO_3_ with the concentrations of 0.00185 and 0.000639, respectively, and the minimum concentration species from the As (III) species in the samples of Karachi and Shikarpur was found for HAsO_3_^−2^ with the concentrations of 3.60 × 10^−22^ and 1.45 × 10^−13^, respectively. Arsenite primarily exists in solution as (H_3_AsO_3_) arsenous acid, which can dissociate into various species depending on the pH of the environment.

In ALF, which has a lower pH (around 4.5) compared to neutral solutions, the dominance of H_3_AsO_3_ and related As(III) species in ALF indicates that arsenic is relatively mobile and highly toxic, because these forms are more soluble, less strongly adsorbed to particle surfaces, and intrinsically more toxic than As(V) species. Under acidic ALF conditions, the coexistence of H3AsO_3_ with H2AsO_4_ suggests that a substantial fraction of arsenic remains in the more toxic trivalent form while also being sufficiently dissolved to be bioaccessible in the lung environment. The predominance of H_3_AsO_3_ in ALF for both Karachi and Shikarpur samples therefore implies a higher toxic potential, even if total arsenic concentrations are similar, because a larger fraction is present as the more harmful trivalent species.

PHREEQC calculations for Pb speciation showed that the predominant species in ALF for the samples of the study areas of Karachi and Shikarpur were Pb(OH)^+^, Pb(OH)_4_^−2^, Pb^+2^, and Pb_3_(OH)_4_^+2^. Moreover, PHREEQC calculations showed the formation of Pb hydroxyl species at low pH conditions, which endorses the findings of a previous study [49]. PHREEQC results suggest that among the species of Pb, the most abundant species found in the samples of both cities was Pb(OH)_4_^−2^. This result is consistent with the findings of the previous study related to the mobilization of Pb [50]. Lead hydroxide can dissociate in solution, particularly in the presence of protons (H^+^), which is relevant in the acidic conditions of ALF (pH around 4.5).

The dominance of Pb(OH)_4_^2−^, Pb(OH)^+^, Pb^2+^, and Pb_3_(OH)_4_^2+^ in ALF shows that lead is present as soluble, labile inorganic complexes that can remain mobile in the acidic ALF environment and contribute significantly to bioaccessible, toxic Pb(II) exposure. Under low pH, hydroxylated Pb(II) complexes and free Pb^2+^ are compatible with relatively high dissolution of Pb-bearing particles in ALF, which supports efficient transport in lung fluids and uptake at cell membranes. Therefore, the dominance of hydroxylated, dissolved Pb(II) species in ALF modeling implies that Pb species associated with PM_2.5RD_ from Karachi and Shikarpur can be efficiently released into lung fluids and present a significant toxic risk, even if total particulate Pb appears moderately bound in the PM_2.5RD_.

The PHREEQC computed values for Cr(III) showed that the predominant species in ALF for the samples of the study areas of Karachi and Shikarpur were Cr^+3^, Cr(OH)^+2^, CrH_2_PO_4_^+2^, and Cr(OH)_3_, whereas from Cr(VI), the dominant species found in ALF by PHREEQC calculation were HCrO_4_^−^, CrO_3_HPO_4_^−2^, CrO_4_^−2^, and H_2_CrO_4_. The results of PHREEQC showed that among the species of Cr(III), the most abundant species found in the samples of both cities was Cr^+3^, whereas among the species of Cr(VI), CrO_4_^−2^ and H_2_CrO_4_ were found dominant in the samples of the study areas of Karachi and Shikarpur, respectively. The dominance of aqueous Cr^3+^ and Cr(VI) oxy-anions in ALF indicates that chromium is present in mobile and bioaccessible forms, and could be associated with Cr(VI) species (HCrO_4_^−^, CrO_4_^2−^, H_2_CrO_4_, CrO_3_HPO_4_^2−^) being far more toxic and generally more mobile than Cr(III) species under the acidic conditions of ALF. Cr(III) in forms such as Cr^3+^, Cr(OH)^2+^, CrH_2_PO_4_^2+^, and Cr(OH)_3_ is less toxic and tends to be less mobile than Cr(VI), but soluble Cr^3+^ in ALF still represents a bioavailable fraction able to interact with cells. Cr(VI) oxy-anions are also much more toxic and carcinogenic than Cr(III), entering cells through anion transporters and then being reduced intracellularly to Cr(III), with intermediate reactive species causing oxidative stress, DNA damage, and mutagenesis. The PHREEQC results suggest that CrO_4_^2−^ is dominant for Karachi samples and H_2_CrO_4_ for Shikarpur samples, showing that Cr(VI) remains abundant in these mobile oxy-anionic forms in ALF, which strongly elevates potential toxicity even if the total Cr amount is the same as Cr(III).

The dominance of arsenic (III) and chromium (VI) species in ALF raises significant toxicological concerns due to their high solubility and bioaccessibility under acidic physiological conditions. Arsenic (III), predominantly existing as arsenious acid (H_3_AsO_3_), can easily permeate cellular membranes and bind to thiol-containing proteins, disrupting enzymatic activities and impairing mitochondrial function. This can provoke oxidative stress, genotoxicity, and apoptosis in exposed cells. Moreover, chromium (VI) species such as hydrogen chromate (HCrO_4_^−^), dichromate (CrO_4_^2−^), hydrochromate (H_2_CrO_4_), and phosphate-bound chromate (CrO_3_HPO_4_^2−^) are potent oxidizing agents that can penetrate cell membranes via anion transporters. Once internalized, these species undergo reductive transformations to chromium (III), generating reactive oxygen species (ROS) and inducing oxidative DNA damage, mutagenesis, and lipid peroxidation. The combined presence of As(III) and Cr(VI) compounds may lead to synergistic toxic interactions, intensifying cellular stress responses and inflammatory pathways. Therefore, evaluating the stability and reactivity of these species in ALF is crucial for understanding their potential health hazards following inhalation of hazardous PM_2.5RD_.

The saturation index (SI) for relevant minerals in the samples of the study areas of Karachi and Shikarpur is illustrated in Table 8 and Table 9, respectively. Mineral precipitation is indicated by a positive SI, whereas mineral solubilization is shown by a negative SI. The values of saturation index results for all the samples of Karachi and Shikarpur were found to be undersaturated with respect to arsenolite and As_2_O_5_. The calculated SI values for Cr minerals, including Cr(OH)_2_, Cr(OH)_3_, Cr_2_O_3_, CrCl_2_, CrCl_3_, and Cr metal, in all the samples of Karachi were found to be undersaturated except the values of the K.O2, K.S2, and K.R1 samples, which were found supersaturated with respect to Cr_2_O_3_. The estimated SI values for the series of Cr minerals, including Cr(OH)_2_, Cr(OH)_3_, Cr_2_O_3_, CrCl_2_, CrCl_3_, and Cr metal, in all the samples of Shikarpur were found to be undersaturated. Chromium’s status as the most bioaccessible metal in Karachi and Shikarpur samples heightens health risks, due to its classification as a known human carcinogen, particularly in its hexavalent Cr(VI) form, which readily enters cells and causes DNA damage. Undersaturation of most Cr minerals, e.g., Cr(OH)_2_, Cr(OH)_3_, across samples promotes dissolution, amplifying threats in high-exposure areas like Karachi, where supersaturation of Cr_2_O_3_ in K.O2, K.S2, and K.R1 offers limited protection by precipitation.

Super saturation of Cr_2_O_3_ in the selected samples from the areas of Karachi implies reduced solubility there, potentially lowering local bioaccessibility and immediate risks compared to fully undersaturated Shikarpur samples. However, overall Cr dominance is the most bioaccessible metal in urgent need of remediation, as even partial supersaturation does not mitigate the carcinogenic hazard from mobile fractions. The results of this investigation are consistent with those of a prior study, which revealed comparable SI values for different Cr compounds [51]. Except for the values of Pb_3_(AsO_4_)_2_, Pb_3_(PO_4_)_2_, and PbHPO_4_ minerals, which demonstrate oversaturation, practically all Pb minerals have negative SI findings, indicating their solubilization under the current experimental conditions. Super saturation of specific Pb minerals Pb_3_(AsO_4_)_2_, Pb_3_(PO_4_)_2_, and PbHPO_4_ and selected Cr minerals such as Cr_2_O_3_ in K.O2, K.S2, and K.R1 implies localized precipitation potential under study conditions, suggesting geochemical controls like high pH or phosphate/arsenate levels trap these heavy metals. This mitigates their solubilization and bioaccessibility compared to undersaturated counterparts, indicating lower immediate bioavailability risks for those samples. However, precipitates could still pose long-term concerns if remobilized by environmental changes.

### 3.5. Comparison of PHREEQC Modeling Results for Data from Two Cities

The geochemical modeling results for arsenic (As), lead (Pb), and chromium (Cr) in the PM_2.5RD_ samples from Karachi and Shikarpur reveal significant differences in metal speciation and concentrations between the two cities. Predominant species in the PM_2.5RD_ samples from Karachi were As(III) and As(V), with arsenite (H_3_AsO_3_) being the most concentrated species (maximum 1.85 × 10^−3^ mol L^−1^). The most stable arsenic forms under the measured low pH conditions were H_3_AsO_3_ and H_2_AsO_4_−, suggesting enhanced arsenic mobilization in residential samples. On the other hand, in the samples from Shikarpur, both As(III) and As(V) were present, but H_3_AsO_3_ concentrations were lower (max 6.39 × 10^−4^ mol L^−1^) than in Karachi, indicating reduced mobilization potential compared with Karachi. Both cities show redox coexistence of As(III) and As(V), but Karachi’s higher fraction of soluble arsenite implies greater environmental mobility and potentially higher exposure risk; both cities were undersaturated with respect to arsenolite and As_2_O_5_, indicating low precipitation potential for arsenic minerals under the modeled conditions.

The dominant lead aqueous species in the samples of Karachi was Pb(OH)_4_^−2^. Saturation indices were generally negative for lead minerals, although a few samples suggested oversaturation for specific lead–arsenate phases. For the samples of Shikarpur, Pb(OH)_4_^−2^ was likewise the predominant aqueous lead species. Overall saturation indices were similarly negative except for isolated oversaturation cases reported in the data. The consistent dominance of Pb(OH)_4_^−2^ in both cities indicates lead is present primarily as hydroxyl complexes under the sampled pH/chemistry, and the generally negative saturation indices point toward lead remaining in solution rather than forming abundant stable mineral phases (with limited, site-specific exceptions). These speciation results imply moderate solubility and transport potential for Pb in PM_2.5_-derived leachates.

The results mirrored those of Karachi: for Cr(III), Cr^3+^ was the most abundant species; for Cr(VI), CrO_4_^−2^ was dominant, with H_2_CrO_4_ also appearing in the Cr(VI) distribution under some conditions. Some Karachi samples showed supersaturation for specific chromium minerals (noted in the original outputs as K.O2, K.S2, and K.R1), suggesting potential for mineral formation in certain microenvironments. Cr(III) is generally considered less toxic and can be an essential nutrient. Its stability in lower pH environments makes it less mobile compared to Cr(VI), which is significant for environmental assessments in water systems [52]. For Shikarpur, Cr^3+^ remained predominant. Cr(III) species and H_2_CrO_4_ were prevalent among Cr(VI) species in the available runs, while saturation indices for chromium compounds were generally negative (undersaturated) across Shikarpur samples. The partitioning between Cr(III) and Cr(VI) differs by oxidation state rather than city, but Karachi shows a greater presence of soluble Cr(VI) oxyanions and localized supersaturation for some Cr minerals, implying a higher potential for chromium mobility and associated risk there. The pH strongly controls the Cr(VI) speciation, consistent with the modeled distributions.

### 3.6. Heavy Metals Speciation Modeling Results by MINTEQ

**Figure 10:**
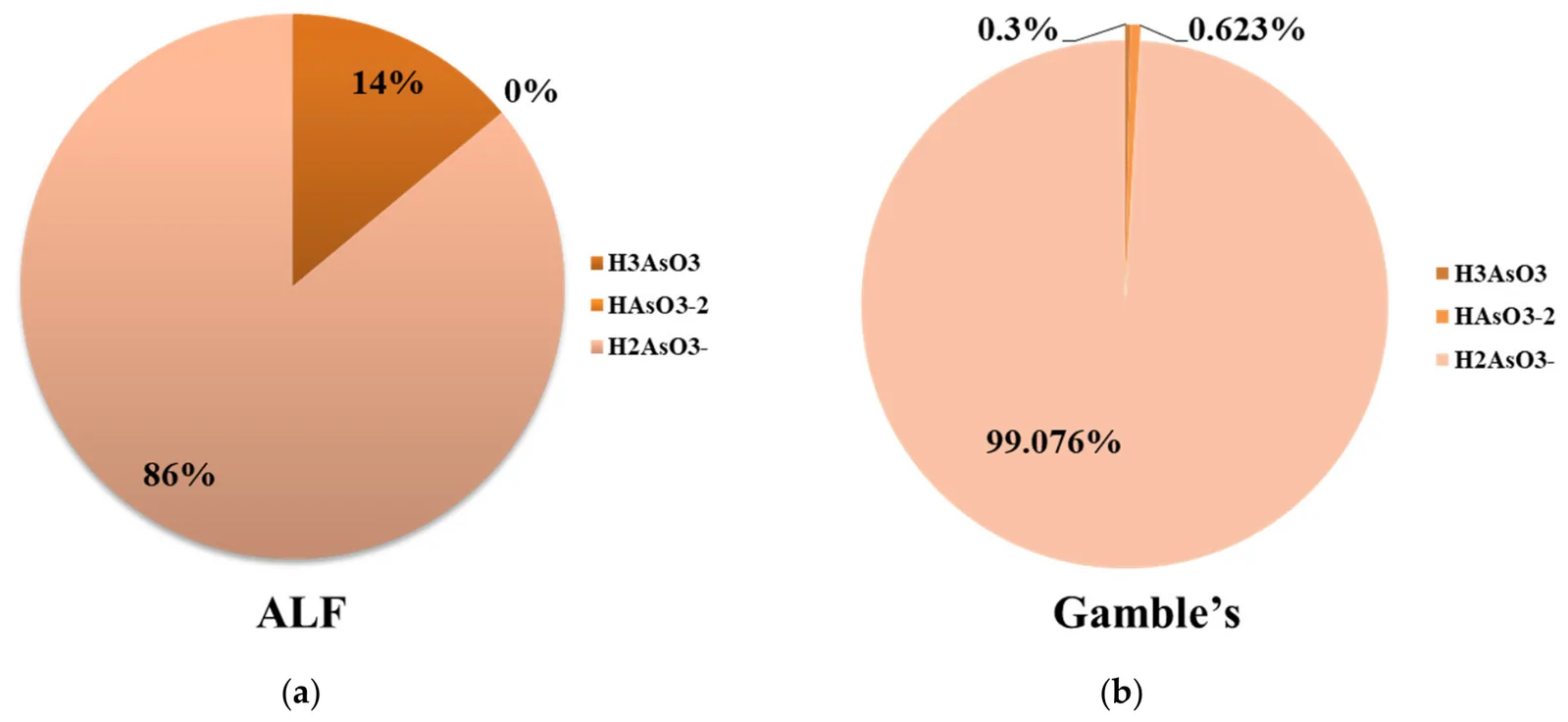
Visual MINTEQ 3.1 speciation modeling of As(III) in contact with (**a**) artificial lysosomal fluid (ALF) and (**b**) Gamble’s solution.

**Figure 11:**
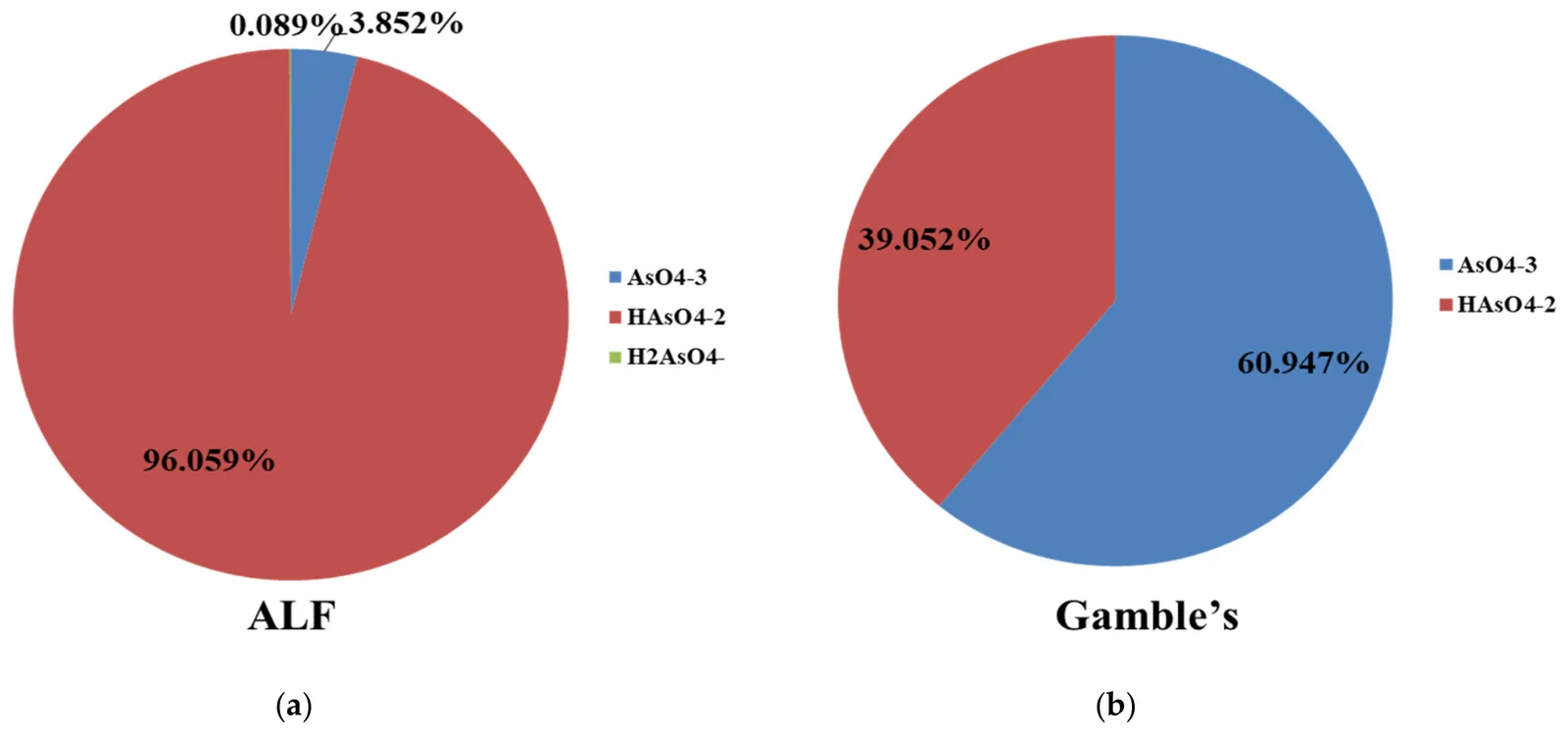
Visual MINTEQ 3.1 speciation modeling of As(V) in contact with (**a**) artificial lysosomal fluid (ALF) and (**b**) Gamble’s solution.

The speciation results simulated through MINTEQ for As showed that As was found in ALF and Gamble’s solution in both oxidation states, As(III) and As(V). The simulation results showed that the highest percent of As(III) was found in both fluids in the form of dihydrogen arsenite (H_2_AsO_3_^−^) (Figure 10). These findings are consistent with findings of previous research, which states that the values of the species of As(III), particularly H_2_AsO_3_^−^, were almost unchanged with the variation in pH until the limit of 8.0 [53]. The MINTEQ speciation results for As(V) showed that more than 95% of As(V) was in the HAsO_4_^−2^ form at pH 4.5 in ALF and more than 60% of As(V) was in the HAsO_4_^−2^ form at pH 7.4 in Gamble’s solution (Figure 11). Hence, the obtained values showed that the speciation of the As(V) form is dependent on the pH conditions.

**Figure 12:**
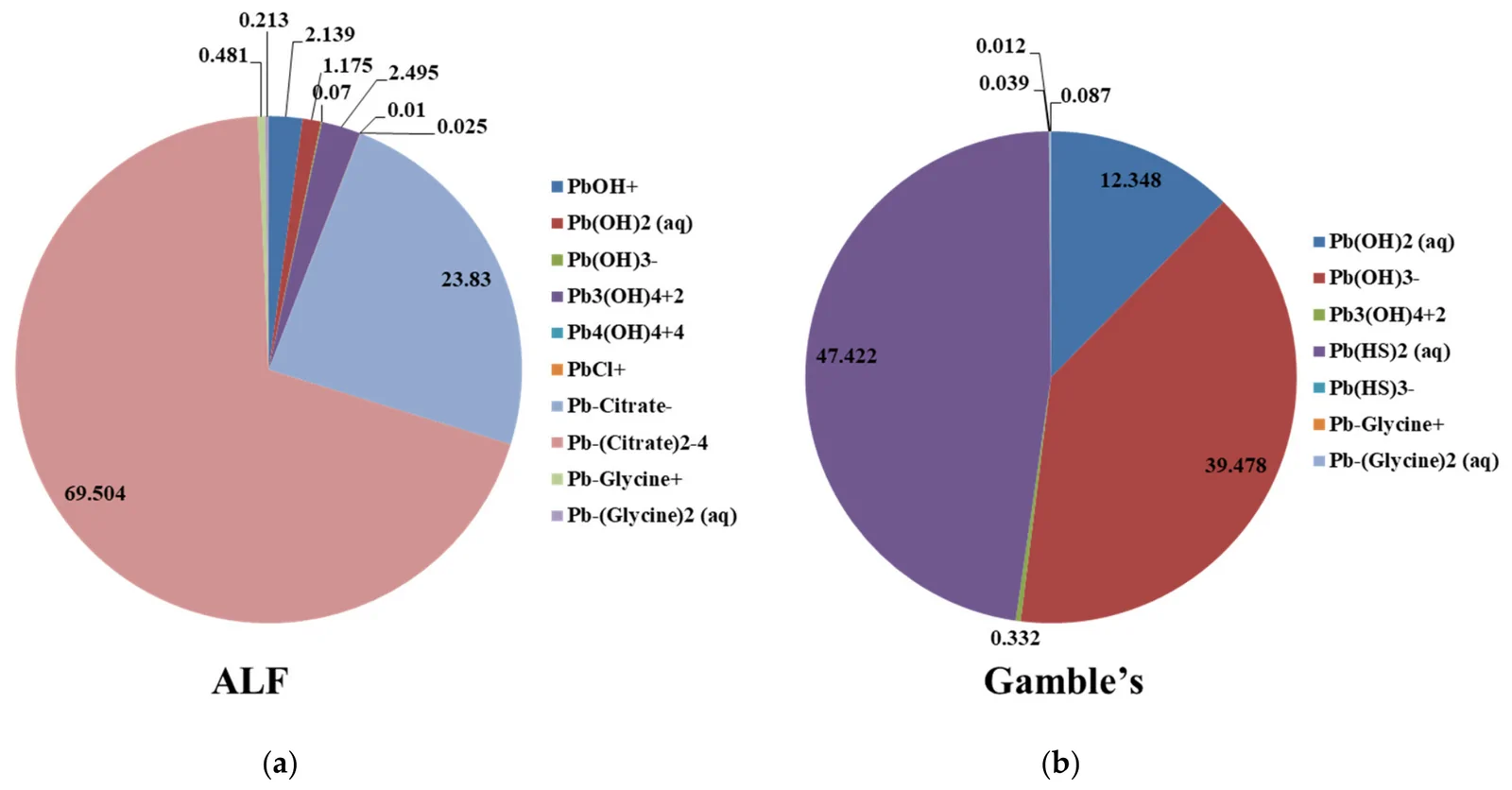
Visual MINTEQ 3.1. Speciation modeling of Pb in contact with (**a**) artificial lysosomal fluid and (**b**) Gamble’s solution.

**Figure 13:**
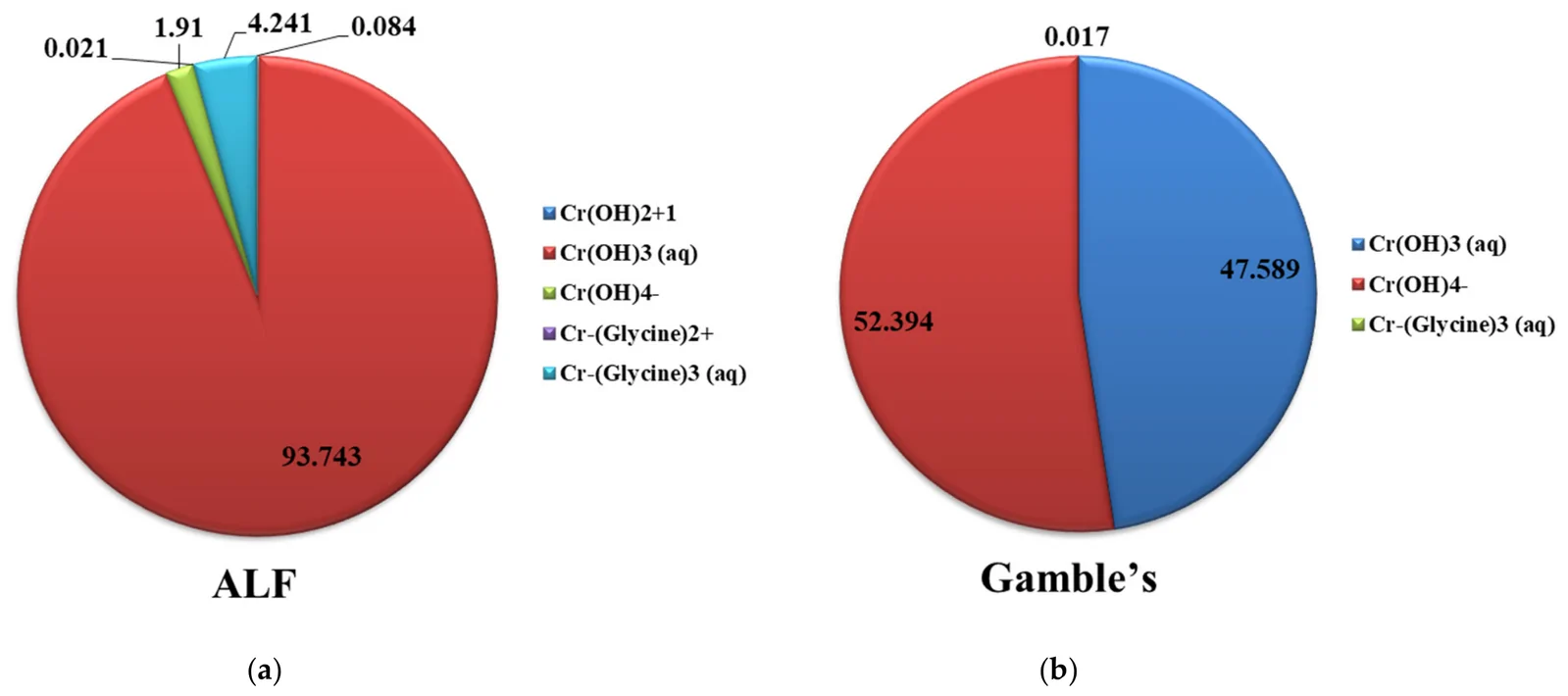
Visual MINTEQ 3.1. Speciation modeling of Cr (III) in contact with (**a**) artificial lysosomal fluid and (**b**) Gamble’s solution.

**Figure 14:**
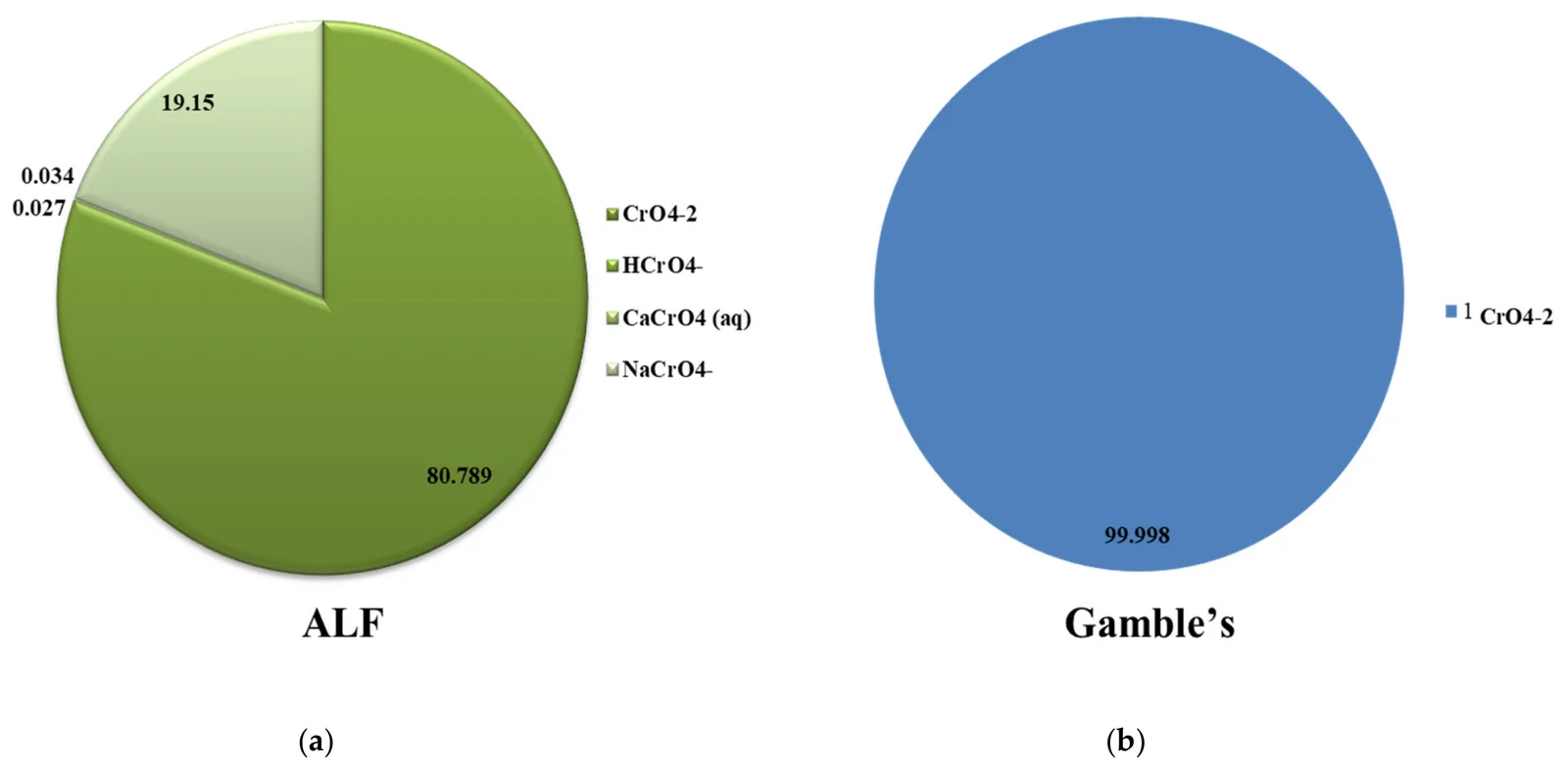
Visual MINTEQ 3.1. Speciation modeling of Cr (VI) in contact with (**a**) artificial lysosomal fluid and (**b**) Gamble’s solution.

The MINTEQ modeling data for Pb showed that the citrate species of Pb were highly soluble under the experimental conditions of ALF, whereas Pb (HS)_2_ was found to be the dominant species of Pb in Gamble’s solution (Figure 12). This result indicates that citrate species are more soluble under low pH conditions. The MINTEQ modeling results for Cr species showed that Cr(OH)_3_ was found to be dominant among the Cr (III) species under the experimental conditions of ALF, whereas Cr(OH)_4_^−^ was found to be dominant among the Cr (III) species in the experimental conditions of Gamble’s solution and Cr(OH)_3_ was the second highest (Figure 13). These outcomes are consistent with the findings of a recent research which states that the amount of Cr(OH)_3_ was gradually decreasing with the increasing amount of pH [54]. Furthermore, the Visual MINTEQ 3.1 speciation modeling results for Cr (VI) showed that among species of Cr (VI), dioxide chromium (CrO_4_^−2^) was found to be the dominant species under both experimental conditions of ALF and Gamble’s solution (Figure 14). The differences in calculated speciation between the two simulated lung fluids likely reflect their distinct pH values and chemical compositions, which alter complexation and aqueous stability and therefore lead to different dominant species for As, Pb, and Cr; accordingly, these outputs should be interpreted as model-based predictions of metal behavior under contrasting lung-fluid conditions rather than direct evidence of species present in the dust.

### 3.7. Comparison of Saturation Index Results of Mineral Speciation in ALF and GS Using MINTEQ 3.1

According to saturation index results calculated by Visual MINTEQ 3.1, As is mainly present as arsenolite, As_2_O_5_(s), and As_2_S_3_. Among them, arsenolite and arsenic sulphide belong to As(III), and As_2_O_5_(s) belongs to As(V). SI values for all arsenic species indicated undersaturation in both ALF and Gamble’s solution. It can be seen from Table 10 that for species of arsenic, saturation index values are increasing with decreasing pH. In this respect, minimum and maximum SI were observed at pH 7 and pH 4.

The values of SI showed supersaturation for Pb minerals including lead (II) hydroxide (Pb(OH)_2_(s)), Pb_2_(OH)_3_Cl(s), Pb_3_(AsO_4_)_2_, Pb_3_(PO_4_)_2_, PbCrO_4_, and PbHPO_4_, and undersaturated values for Pb_2_O(OH)_2_ and PbO:0.3H_2_O under the experimental conditions of ALF. Whereas under the experimental conditions of Gamble’s solution, the values of SI showed undersaturation for Pb minerals including Pb_3_(AsO_4_)_2_, PbCrO_4_, and PbHPO_4_ except Pb(OH)_2_, Pb_2_O(OH)_2_, Pb_3_(PO_4_)_2_, and PbO:0.3H_2_O, whose values were determined as supersaturated. The saturation index for the Pb species was both positive and negative in ALF and Gamble’s solution, which endorses the findings of previous research [55]. For the chromium SI analysis, four different types of chromium minerals were identified, including Cr(OH)_3_, Cr_2_O_3_, CrCl_3_, and CrO_3_ under the experimental conditions of ALF; among them, the SI values of Cr(OH)_3_ and Cr_2_O_3_ revealed supersaturation, whereas CrCl_3_ and CrO_3_ showed undersaturated values. In Gamble’s solution, SI analysis has determined three types of Chromium minerals, including Cr(OH)_3_, Cr_2_O_3_, and CrO_3_. The SI values of Cr(OH)_3_ and Cr_2_O_3_ indicated supersaturation, whereas the SI values of CrO_3_ showed undersaturation. Cr(OH)_3_ and Cr_2_O_3_ were the stable species with a saturation index larger than zero, which is consistent with a recent study’s findings [56].

## 4. Conclusions

An in vitro bioaccessibility test was conducted on PM_2.5RD_ samples using artificial lysosomal fluid (ALF). During the inhalation bioaccessibility test, the bioaccessibility of only three potential toxic elements, including As, Pb, and Cr, was found beyond the detection limits. The present study has also revealed that among all three detected heavy metals, Cr, which is a well-known carcinogenic element, has shown the highest absorbing capacity during the inhalation bioaccessibility test. Assessment of the pollution level of heavy metals in the PM_2.5RD_ samples using contamination factor (CF), integrated pollution index (IPI), pollution load index (PLI), and potential ecological risk index has shown extreme levels of pollution by Cu, Pb, Cd, Zn, and Sb in all four areas of Karachi and Shikarpur. Two prominent geochemical modeling programs, PHREEQC and Visual MINTEQ, were used to investigate geochemical behavior, speciation, and saturation indices of relevant heavy metals. This study confirmed the presence of several species of As, Pb, and Cr under the given experimental conditions of two different simulated lung fluids: ALF and Gamble’s solution. According to Visual MINTEQ results, the bioaccessibility of metal(loid)s in PM_2.5RD_ samples is influenced not only by the synthetic fluid’s concentration and pH, but also by their speciation. Furthermore, the findings of the Visual MINTEQ simulation revealed that maximum saturation as a function of pH occurs at pH values ranging from 4.5 to 7.4. Lastly, road dust, particularly PM_2.5RD_, was identified as the prominent source of As, Pb, and Cr exposure for the urban population. It is also strongly recommended that careful consideration should be paid to road emissions in residential, school, and hospital areas in order to protect at-risk communities from the adverse effects of heavy metals, in particular the known carcinogen Cr. To mitigate exposure risks, priority should be given to implementing dust control strategies in sensitive zones such as schools, hospitals, and residential areas near emission sources. Measures such as regular surface cleaning, improved ventilation, and vegetation buffers can effectively reduce PM_2.5RD_ dispersion. These interventions should be supported by strict policy enforcement and community awareness programs. Future studies are encouraged to integrate metal bioaccessibility data with in vivo models or epidemiological datasets to elucidate exposure–health relationships, thereby improving risk assessment precision and informing sustainable urban environmental management frameworks.

## Figures and Tables

**Table 1:** Heavy metal concentration (mg/kg) in PM_2.5RD_ fraction of road dust of all four areas of Karachi. Reproduced from [Haseeb Tufail Moryani], International Journal of Environmental Research and Public Health 2020, 17, 7124 [25].

Statistical Values		Concentration (mg/kg)						
	As	Cu	Pb	Zn	Cd	Ni	Sb	Cr
Minimum	35.1	70.9	140.5	1332.3	17.9	142.6	24.4	57.6
Maximum	228	913.3	712.3	8299	114.1	689	122.5	326.3
Mean	127.7	332.9	426.6	4254.4	62.3	389.7	70.4	148.1
S.D	34.1	347.4	220.7	2853.5	40.4	219.2	36	98.8

**Table 2:** Heavy metal concentration (mg/kg) in PM_2.5RD_ fraction of road dust of all four areas of Shikarpur. Reproduced from [Haseeb Tufail Moryani], International Journal of Environmental Research and Public Health 2020, 17, 7124 [25].

Statistical Values		Concentration (mg/kg)						
	As	Cu	Pb	Zn	Cd	Ni	Sb	Cr
Minimum	86.1	111.7	331.4	4726.3	39.6	57.9	150.5	171.1
Maximum	197.6	507.6	886.2	11,683.9	78.2	449.4	473.2	744.0
Mean	135.8	245.8	538.4	8351.0	57.6	131.7	314.5	346.6
S.D	34.4	243.0	611.3	3505.3	20.5	216.0	123.2	352.1

**Table 4:** Results of potential ecological risk assessment for heavy metals in Karachi.

	Er						RI
	Cu	Pb	Ni	Cd	Cr	Zn	
Mean	36.98	102.46	17.19	6232.52	3.29	44.78	8582.99
Maximum	101.48	178.09	30.40	11,411.94	3.94	87.35	49,860.18
Minimum	7.88	35.14	6.29	1793.84	1.28	14.02	26.33

**Table 5:** Results of potential ecological risk assessment for heavy metals in Shikarpur.

	Er						RI
	Cu	Pb	Ni	Cd	Cr	Zn	
Mean	27.31	134.60	5.81	5763.62	7.70	87.90	8035.95
Maximum	56.40	221.55	19.82	7825.58	16.53	120.88	46,108.97
Minimum	12.41	104.76	2.55	3963.04	3.80	49.75	46.50

**Table 6:** Concentrations (M) of dissolved As, Pb, and Cr for the studied sites of Karachi in ALF using PHREEQC (mol/L).

Site	As		Pb	Cr	
	As (III)	As (V)		Cr (III)	Cr (VI)
K.R (1)	6.82 × 10^−3^	0.2315	0.01501	0.1839	2.802 × 10^−31^
K.R (2)	4.17 × 10^−3^	0.1330	0.9363	0.1661	3.634 × 10^−31^
K.S (1)	3.10 × 10^−3^	0.09649	0.3401	0.1165	7.613 × 10^−31^
K.S (2)	1.17 × 10^−3^	0.03505	0.1488	0.05939	1.626 × 10^−30^
K.H (1)	5.56 × 10^−3^	0.1831	0.1323	0.3410	3.673 × 10^−32^
K.H (2)	3.10 × 10^−3^	0.09673	3.755	0.09570	1.031 × 10^−30^
K.O (1)	5.10 × 10^−3^	0.1661	8.04 × 10^−6^	0.1671	3.581 × 10^−31^
K.O (2)	2.83 × 10^−3^	0.08764	0.2624	0.09936	9.788 × 10^−31^

**Table 7:** Concentrations (M) of dissolved As, Pb, and Cr for the studied sites of Shikarpur in ALF using PHREEQC (mol/L).

Site	As		Pb	Cr	
	As (III)	As (V)		Cr (III)	Cr (VI)
S.R (1)	1.50 × 10^−3^	0.04812	187.6	0.2323	1.589 × 10^−29^
S.R (2)	4.05 × 10^−4^	0.1757	0.4865	0.3264	1.087 × 10^−33^
S.S (1)	8.18 × 10^−5^	0.01362	0.0534	7.36 × 10^−4^	1.950 × 10^−35^
S.S (2)	6.30 × 10^−4^	0.1015	2.66 × 10^−5^	0.2911	8.034 × 10^−33^
S.H (1)	2.18 × 10^−5^	0.1001	0.3852	0.8648	6.457 × 10^−35^
S.H (2)	5.58 × 10^−5^	0.1795	0.6351	0.6371	7.939 × 10^−35^
S.O (1)	1.36 × 10^−4^	0.2106	0.7946	0.409	1.529 × 10^−34^
S.O (2)	5.33 × 10^−4^	0.1377	0.5621	0.1997	2.064 × 10^−33^

**Table 8:** Calculated saturation index for the samples of Karachi using PHREEQC and database MINTEQ.v4.dat.

Species	K.R (1)	K.R (2)	K.S (1)	K.S (2)	K.H (1)	K.H (2)	K.O (1)	K.O (2)
As minerals								
Arsenolite	−9.16	−9.73	−9.65	−8.55	−12.79	−44.07	−9.84	−9.11
As_2_O_5_	−14.42	−14.70	−14.66	−14.11	−16.24	−31.87	−14.76	−14.39
Cr minerals								
Cr(OH)_2_	−17.44	−19.09	−18.55	−13.71	−21.50	−25.06	−19.56	−15.85
Cr(OH)_3_	−2.49	−4.14	−3.60	1.24	−6.56	−10.11	−4.61	−0.90
Cr_2_O_3_	0.01	−3.29	−2.22	7.46	−8.11	−15.23	−4.24	3.18
CrCl_2_	−32.64	−33.33	−32.97	−28.47	−35.22	−41.91	−33.69	−30.82
CrCl_3_	−34.31	−34.52	−34.25	−29.92	−36.15	−44.40	−34.82	−32.37
Crmetal	−53.16	−54.82	−54.28	−49.44	−57.22	−60.78	−55.29	−51.58
CrO_3_	−39.29	−40.94	−40.40	−35.56	−43.35	−46.91	−41.41	−37.70
Pb minerals								
Pb(OH)_2_	−0.71	−1.90	−1.59	−0.74	−3.06	−8.34	−2.08	−0.67
Pb_2_(OH)_3_Cl	−0.93	−2.82	−2.30	−0.76	−4.89	−17.01	−3.13	−0.73
Pb_2_O(OH)_2_	−12.10	−14.47	−13.86	−12.15	−16.80	−27.35	−14.84	−12.02
Pb_2_O_3_	−29.95	−32.32	−31.72	−30.01	−34.65	−45.20	−32.69	−29.87
Pb_3_(AsO_4_)_2_	7.48	3.64	4.59	7.70	−1.39	−32.85	3.03	7.62
Pb_3_(PO_4_)_2_	9.86	6.61	7.48	7.03	3.08	−20.32	6.09	9.41
PbCrO_4_	−23.19	−26.02	−25.18	−19.48	−29.60	−38.43	−26.68	−21.56
PbHPO_4_	3.44	2.41	2.70	2.05	1.23	−7.83	2.24	3.20
Pbmetal	−14.19	−15.38	−15.08	−14.23	−16.54	−21.82	−15.57	−14.16

**Table 9:** Calculated saturation index for the samples of Shikarpur using PHREEQC and database MINTEQ.v4.dat.

Species	S.R (1)	S.R (2)	S.S (1)	S.S (2)	S.H (1)	S.H (2)	S.O (1)	S.O (2)
As minerals								
Arsenolite	−10.38	−11.17	−13.93	−10.40	−16.23	−14.60	−13.06	−10.70
As_2_O_5_	−15.03	−15.42	−16.81	−15.17	−17.96	−17.14	−16.37	−15.19
Cr minerals								
Cr(OH)_2_	−20.13	−20.26	−21.58	−18.24	−22.30	−22.11	−21.59	−19.78
Cr(OH)_3_	−5.19	−5.31	−6.64	−3.76	−7.36	−7.17	−6.65	−4.84
Cr_2_O_3_	−5.38	−5.63	−8.27	−2.52	−9.72	−9.34	−8.30	−4.68
CrCl_2_	−34.61	−34.19	−38.69	−35.35	−35.87	−35.74	−35.30	−33.86
CrCl_3_	−35.92	−35.22	−41.31	−38.43	−36.73	−36.62	−36.22	−34.96
Crmetal	−55.86	−55.98	−57.30	−54.13	−58.02	−57.83	−57.31	−55.51
CrO_3_	−41.98	−42.11	−43.43	−41.25	−44.16	−43.97	−43.45	−41.64
Pb minerals								
Pb(OH)_2_	−2.91	−2.52	−2.23	−4.14	−3.73	−3.48	−3.10	−2.17
Pb_2_(OH)_3_Cl	−4.97	−3.90	−4.93	−8.73	−6.16	−5.68	−4.97	−3.29
Pb_2_O(OH)_2_	−16.49	−15.71	−15.14	−18.95	−18.14	−17.63	−16.88	−15.02
Pb_2_O_3_	−34.35	−33.56	−32.99	−36.64	−35.99	−35.48	−34.73	−32.87
Pb_3_(AsO_4_)_2_	0.28	1.06	0.54	−3.55	−5.12	−3.54	−1.64	2.32
Pb_3_(PO_4_)_2_	2.54	4.77	5.31	−2.74	0.90	1.73	2.95	5.82
PbCrO_4_	−28.08	−27.81	−28.85	−28.58	−31.07	−30.63	−29.73	−26.99
PbHPO_4_	0.88	1.80	1.92	−1.15	0.47	0.76	1.18	2.15
Pbmetal	−16.39	−16.00	−15.71	−17.79	−17.21	−16.96	−16.58	−15.66

**Table 10:** Calculated saturation index of mineral speciation in ALF and GS using MINTEQ 3.1.

The Presence of As			The Presence of Pb			The Presence of Cr		
Mineral	SI(ALF)	SI(GS)	Mineral	SI(ALF)	SI(GS)	Mineral	SI(ALF)	SI(GS)
Arsenolite	−6.418	−9.855	Pb(OH)_2_	4.449	5.437	Cr(OH)_3_ (am)	3.969	3.637
As_2_O_5_	−34.154	−41.857	Pb_2_(OH)_3_Cl	4.636		Cr_2_O_3_	8.173	7.492
As_2_S_3_		−15.455	Pb_2_O(OH)_2_	−1.778	0.192	CrCl_3_	−43.858	
			Pb_3_(AsO_4_)_2_	3.306	−1.451	CrO_3_	−19.568	−22.932
			Pb_3_(PO_4_)_2_	9.481	8.29			
			PbCrO_4_	1.697	−0.686			
			PbHPO_4_	0.672	−0.411			
			PbO:0.3H_2_O	−0.773	0.211			

## Data Availability

The datasets used and/or analyzed during the current study are available from the corresponding author on reasonable request.

## Supplementary Materials

The following supporting information can be downloaded at https://www.mdpi.com/article/10.3390/toxics14080704/s1, Table S1. Sampling locations and characteristics; Table S2. Composition of artificial lysosomal fluid (ALF); Table S3. Value ranges and pollution levels description of contamination factor; Table S4. Value ranges and pollution levels description IPI, and PLI [2]; Table S5. Grades for ecological risk factor of individual metal; Table S6. Grades of ecological risk index of heavy metal pollution; Table S7. Distribution of species for leachates in the samples of Karachi city; Table S8. Distribution of species for leachates in the samples of Shikarpur city. Data calculated using PHREEQC and database MINTEQ. Values in molality Data calculated using PHREEQC and database MINTEQ. Values in molality [28,57,58,59].

## References

[1] Sheng M.S., Wen L., Le Truong Q., Davy P. (2025). Impact of road dust on local air pollution: A case study of Auckland, New Zealand. J. R. Soc. N. Z..

[2] Alves C.A., Evtyugina M., Vicente A.M.P., Vicente E.D., Nunes T.V., Silva P.M.A., Duarte M.A.C., Pio C.A., Amato F., Querol X. (2018). Chemical profiling of PM10 from urban road dust. Sci. Total Environ..

[3] Chen S., Zhang X., Lin J., Huang J., Zhao D., Yuan T., Huang K., Luo Y., Jia Z., Zang Z. (2019). Fugitive Road Dust PM 2.5 Emissions and Their Potential Health Impacts. Environ. Sci. Technol..

[4] Shahab A., Hui Z., Rad S., Xiao H., Siddique J., Huang L., Ullah H., Rashid A., Taha M., Zada N. (2022). A comprehensive review on pollution status and associated health risk assessment of human exposure to selected heavy metals in road dust across different cities of the world. Environ. Geochem. Health.

[5] Lough G.C., Schauer J.J., Park J.-S., Shafer M.M., DeMinter J.T., Weinstein J.P. (2005). Emissions of Metals Associated with Motor Vehicle Roadways. Environ. Sci. Technol..

[6] Lurie K., Nayebare S., Fatmi Z., Carpenter D., Siddique A., Malashock D., Khan M., Zeb J., Hussain M., Khatib F. (2019). PM2.5 in a Megacity of Asia (Karachi): Source Apportionment and Health Effects. Atmos. Environ..

[7] Kastury F., Smith E., Juhasz A.L. (2017). A critical review of approaches and limitations of inhalation bioavailability and bioaccessibility of metal(loid)s from ambient particulate matter or dust. Sci. Total Environ..

[8] Khan R., Strand M. (2018). Road Dust and It’s Effect on Human Health: A literature Review. Epidemiol. Health.

[9] Andrade-Oliva M.L.A., Barbosa-Sánchez A.L., Márquez-Herrera C.E., Aztatzi-Aguilar O.G., Debray-García Y., Sierra-Vargas M.P. (2025). Bioaccessibility of metal (loid)s from fine particulate matter (PM(2.5)) varies by artificial lung fluid type, season, and location in Mexico City. Environ. Sci. Pollut. Res. Int..

[10] Rahman M., Khan H., Jolly Y., Kabir J., Akter S., Salam A. (2019). Assessing risk to human health for heavy metal contamination through street dust in the Southeast Asian Megacity: Dhaka, Bangladesh. Sci. Total Environ..

[11] Hernández-Pellón A., Nischkauer W., Limbeck A., Fernández-Olmo I. (2018). Metal(loid) bioaccessibility and inhalation risk assessment: A comparison between an urban and an industrial area. Environ. Res..

[12] Sánchez-Piñero J., Novo-Quiza N., Pernas-Castaño C., Moreda-Piñeiro J., Muniategui-Lorenzo S., López-Mahía P. (2022). Inhalation bioaccessibility of multi-class organic pollutants associated to atmospheric PM2.5: Correlation with PM2.5 properties and health risk assessment. Environ. Pollut..

[13] Xie S.-Y., Lao J.-Y., Wu C.-C., Bao L.-J., Zeng E.Y. (2018). In vitro inhalation bioaccessibility for particle-bound hydrophobic organic chemicals: Method development, effects of particle size and hydrophobicity, and risk assessment. Environ. Int..

[14] Mukhtar A., Limbeck A. (2013). Recent developments in assessment of bio-accessible trace metal fractions in airborne particulate matter: A review. Anal. Chim. Acta.

[15] Kastury F., Smith E., Karna R., Scheckel K., Juhasz A. (2018). Methodological factors influencing inhalation bioaccessibility of metal(loid)s in PM 2.5 using simulated lung fluid. Environ. Pollut..

[16] Lu Y., Lin S., Fatmi Z., Malashock D., Hussain M., Siddique A., Carpenter D., Lin Z., Khwaja H. (2019). Assessing the association between fine particulate matter (PM2.5) constituents and cardiovascular diseases in a mega-city of Pakistan. Environ. Pollut..

[17] Hoor-Ul-Ain S. (2019). An empirical review of Karachi’s transportation predicaments: A paradox of public policy ranging from personal attitudes to public opinion in the megacity. J. Transp. Health.

[18] Shafiq M., Iqbal M.Z., Arayne M., Athar M. (2012). Biomonitoring of Heavy Metal Contamination in Pongamia pinnata and Peltophorum pterocarpum Growing in the Polluted Environment of Karachi. J. Appl. Bot. Food Qual..

[19] Khwaja H., Fatmi Z., Malashock D., Aminov Z., Kazi A., Siddique A., Zeb J., Carpenter D. (2012). Effect of air pollution on daily morbidity in Karachi, Pakistan. J. Local Glob. Health Sci..

[20] Kazmi J., Zubair S. (2014). Estimation of Vehicle Damage Cost Involved in Road Traffic Accidents in Karachi, Pakistan: A Geospatial Perspective. Procedia Eng..

[21] Iftikhar M., Alam K., Sorooshian A., Syed W.A., Bibi S., Bibi H. (2018). Contrasting aerosol optical and radiative properties between dust and urban haze episodes in megacities of Pakistan. Atmos. Environ..

[22] Ali M., Adnan M., Noman S., Baqueri S. (2014). Estimation of Traffic Congestion Cost-A Case Study of a Major Arterial in Karachi. Procedia Eng..

[23] Shahab A., Qi S., Rashid A., Hasan F., Sohail M.T. (2016). Evaluation of Water Quality for Drinking and Agricultural Suitability in the Lower Indus Plain in Pakistan. Pol. J. Environ. Stud..

[24] Chandio A.A., Jiang Y., Rehman A. (2018). Credit margin of investment in the agricultural sector and credit fungibility: The case of smallholders of district Shikarpur, Sindh, Pakistan. Financ. Innov..

[25] Moryani H.T., Kong S., Du J., Bao J. (2020). Health Risk Assessment of Heavy Metals Accumulated on PM2.5 Fractioned Road Dust from Two Cities of Pakistan. Int. J. Environ. Res. Public Health.

[26] Othman M., Latif M.T., Matsumi Y. (2019). The exposure of children to PM2.5 and dust in indoor and outdoor school classrooms in Kuala Lumpur City Centre. Ecotoxicol. Environ. Saf..

[27] Tian S., Liang T., Li K. (2019). Fine road dust contamination in a mining area presents a likely air pollution hotspot and threat to human health. Environ. Int..

[28] Midander K., Pan J., Odnevall Wallinder I., Leygraf C. (2007). Metal release from stainless steel particles in vitro—Influence of particle size. J. Environ. Monit..

[29] Mohmand J., Eqani S.A.M.A.S., Fasola M., Alamdar A., Mustafa I., Ali N., Liu L., Peng S., Shen H. (2015). Human exposure to toxic metals via contaminated dust: Bio-accumulation trends and their potential risk estimation. Chemosphere.

[30] Turekian K.K., Wedepohl K.H. (1961). Distribution of the Elements in Some Major Units of the Earth’s Crust. GSA Bull..

[31] Tomlinson D.L., Wilson J.G., Harris C.R., Jeffrey D.W. (1980). Problems in the assessment of heavy-metal levels in estuaries and the formation of a pollution index. Helgol. Meeresunters..

[32] Jahan S., Strezov V. (2018). Comparison of pollution indices for the assessment of heavy metals in the sediments of seaports of NSW, Australia. Mar. Pollut. Bull..

[33] Hettiarachchi E., Paul S., Cadol D., Frey B., Rubasinghege G. (2019). Mineralogy Controlled Dissolution of Uranium from Airborne Dust in Simulated Lung Fluids (SLFs) and Possible Health Implications. Environ. Sci. Technol. Lett..

[34] Li S.-W., Li H.-B., Luo J., Li H.-M., Qian X., Liu M.-M., Bi J., Cui X.-Y., Ma L.Q. (2016). Influence of pollution control on lead inhalation bioaccessibility in PM2.5: A case study of 2014 Youth Olympic Games in Nanjing. Environ. Int..

[35] Wan X., Gu G., Lei M., Zeng W. (2020). Bioaccessibility of metals/metalloids in willow catkins collected in urban parks of Beijing and their health risks to human beings. Sci. Total Environ..

[36] Huang M., Chen X., Zhao Y., Yu Chan C., Wang W., Wang X., Wong M.H. (2014). Arsenic speciation in total contents and bioaccessible fractions in atmospheric particles related to human intakes. Environ. Pollut..

[37] Wiseman C.L.S., Zereini F. (2014). Characterizing metal(loid) solubility in airborne PM10, PM2.5 and PM1 in Frankfurt, Germany using simulated lung fluids. Atmos. Environ..

[38] Zereini F., Wiseman C., Püttmann W. (2012). In Vitro Investigations of Platinum, Palladium, and Rhodium Mobility in Urban Airborne Particulate Matter (PM10, PM2.5, and PM1) Using Simulated Lung Fluids. Environ. Sci. Technol..

[39] Zhong L., Liu X., Hu X., Chen Y., Wang H., Lian H.-Z. (2020). In vitro inhalation bioaccessibility procedures for lead in PM2.5 size fraction of soil assessed and optimized by in vivo-in vitro correlation. J. Hazard. Mater..

[40] Liu Y., Hu J., Wang X., Jia J., Li J., Wang L., Hao L., Gao P. (2021). Distribution, bioaccessibility, and health risk assessment of heavy metals in PM2.5 and PM10 during winter heating periods in five types of cities in Northeast China. Ecotoxicol. Environ. Saf..

[41] Drahota P., Raus K., Rychlíková E., Rohovec J. (2018). Bioaccessibility of As, Cu, Pb, and Zn in mine waste, urban soil, and road dust in the historical mining village of Kaňk, Czech Republic. Environ. Geochem. Health.

[42] Liu J., Zhang A., Chen Y., Zhou X., Zhou A., Cao H. (2021). Bioaccessibility, source impact and probabilistic health risk of the toxic metals in PM2.5 based on lung fluids test and Monte Carlo simulations. J. Clean. Prod..

[43] Wang S., Hu G., Yu R., Shen H., Yan Y. (2021). Bioaccessibility and source-specific health risk of heavy metals in PM2.5 in a coastal city in China. Environ. Adv..

[44] Li L., Xing W., Zhao Q., Scheckel K., Zheng L. (2019). Inhalation bioaccessibility of Cd, Cu, Pb and Zn and speciation of Pb in particulate matter fractions from areas with different pollution characteristics in Henan Province, China. Ecotoxicol. Environ. Saf..

[45] Huang H., Jiang Y., Xu X., Cao X. (2018). In vitro bioaccessibility and health risk assessment of heavy metals in atmospheric particulate matters from three different functional areas of Shanghai, China. Sci. Total Environ..

[46] Guney M., Bourges C., Chapuis R., Zagury G. (2016). Lung bioaccessibility of As, Cu, Fe, Mn, Ni, Pb, and Zn in fine fraction. Sci. Total Environ..

[47] Alsanad A., Alolayan M. (2020). Heavy metals in road-deposited sediments and pollution indices for different land activities. Environ. Nanotechnol. Monit. Manag..

[48] Saeedi M., Li L.Y., Salmanzadeh M. (2012). Heavy metals and polycyclic aromatic hydrocarbons: Pollution and ecological risk assessment in street dust of Tehran. J. Hazard. Mater..

[49] Wang Y.-Y., Chai L.-Y., Chang H., Peng X.-Y., Shu Y.-D. (2009). Equilibrium of hydroxyl complex ions in Pb2+-H2O system. Trans. Nonferrous Met. Soc. China.

[50] Navarro A., Cardellach E., Corbella M. (2011). Immobilization of Cu, Pb and Zn in mine-contaminated soils using reactive materials. J. Hazard. Mater..

[51] Halim C., Short S., Scott J., Amal R., Low G. (2005). Modelling the leaching of Pb, Cd, As, and Cr from cementitious waste using PHREEQC. J. Hazard. Mater..

[52] Dvoynenko O., Lo S.-L., Chen Y.-J., Chen G.W., Tsai H.-M., Wang Y.-L., Wang J.-K. (2021). Speciation Analysis of Cr(VI) and Cr(III) in Water with Surface-Enhanced Raman Spectroscopy. ACS Omega.

[53] Fulladosa E., Murat J.C., Martínez M., Villaescusa I. (2004). Effect of pH on Arsenate and Arsenite Toxicity to Luminescent Bacteria (Vibrio fischeri). Arch. Environ. Contam. Toxicol..

[54] Xia X., Yang J., Yan Y., Wang J., Hu Y., Zeng X. (2020). Molecular Sorption Mechanisms of Cr(III) to Organo-Ferrihydrite Coprecipitates Using Synchrotron-Based EXAFS and STXM Techniques. Environ. Sci. Technol..

[55] Ahmad M., Moon D.H., Lim K.J., Shope C.L., Lee S.S., Usman A.R.A., Kim K.-R., Park J.-H., Hur S.-O., Yang J.E. (2012). An assessment of the utilization of waste resources for the immobilization of Pb and Cu in the soil from a Korean military shooting range. Environ. Earth Sci..

[56] Guo S.-S., Xu Y.-H., Yang J.-Y. (2021). Simulating the migration and species distribution of Cr and inorganic ions from tanneries in the vadose zone. J. Environ. Manag..

[57] Rehman A., Liu G., Yousaf B., Zia-Ur-Rehman M., Ali M.U., Rashid M.S., Farooq M.R., Javed Z. (2020). Characterizing pollution indices and children health risk assessment of potentially toxic metal(oid)s in school dust of Lahore, Pakistan. Ecotoxicol. Environ. Saf..

[58] Mirzaei R., Ghorbani H., Moghaddas N.H., Martín J.A.R. (2014). Ecological risk of heavy metal hotspots in topsoils in the Province of Golestan, Iran. J. Geochem. Explor..

[59] Hakanson L. (1980). An ecological risk index for aquatic pollution control.a sedimentological approach. Water Res..

