# Supplementary material for: Inhalation Bioaccessibility, Pollution Assessment, and Speciation of Heavy Metals in PM2.5RD Road Dust from Urban and Semi-Urban Areas of Pakistan
Source: Toxics. 2026 Aug 10;14(8):704. doi: 10.3390/toxics14080704 (PMC13517932; doi:10.3390/toxics14080704)
Supplement: Supplementary file 1 [file toxics-14-00704-s001.zip › toxics-4448906-supplementary.pdf]

Supplementary material

Table S1 Sampling locations and characteristics.

Sampling city	Abbreviation	Nature of road	Activity
Karachi	K.R1	Major road	Residential
Karachi	K.R2	Gate of residential building along the major road	Residential
Karachi	K.O1	Major road	Offices
Karachi	K.O2	Gate of offices building along the major road	Offices
Karachi	K.H1	Major road	Hospital
Karachi	K.H2	Gate of hospital along the major road	Hospital
Karachi	K.S1	Major road	School
Karachi	K.S2	Gate of school along the major road	School
Shikarpur	S.R1	Major road	Residential
Shikarpur	S.R2	Gate of residential building along the major road	Residential
Shikarpur	S.O1	Major road	Offices
Shikarpur	S.O2	Gate of offices building along the major road	Offices
Shikarpur	S.H1	Major road	Hospital
Shikarpur	S.H2	Gate of hospital along the major road	Hospital
Shikarpur	S.S1	Major road	School
Shikarpur	S.S2	Gate of school along the major road	School

Table S2 Composition of artificial lysosomal fluid (ALF).

**Reference**	**[1]**
**Constituents (g/L)**	**Artificial lysosomal fluid (ALF)**
Sodium chloride	3.21
Sodium hydroxide	6.00
Citric acid	20.8
Calcium chloride (dihydrate)	0.128
Sodium hydrogen phosphate dibasic (anhydrous)	0.071
Sodium sulphate	0.039
Magnesium chloride (anhydrous)	0.05
Glycine	0.059
Sodium citrate (dihydrate)	0.077
Sodium tartrate (dihydrate)	0.09
Sodium lactate	0.085
Sodium pyruvate	0.086
Benzalkonium chloride	0.05

Table S3 Value ranges and pollution levels description of contamination factor.

**Values range**	**Pollution level description**
$PI \leq 1$	Low level of Pollution
$1 < PI \leq 3$	Middle level of Pollution
$PI > 3$	High level of Pollution

Table S4 Value ranges and pollution levels description IPI, and PLI [2]

Index	Value range	Pollution level description
IPI		
	IPI≤1	Low level of Pollution
	1<IPI≤2	Middle level of Pollution
	IPI>2	High level of Pollution
PLI		
	PLI<1	No pollution
	PLI>1	Pollution

Table S5 Grades for ecological risk factor of individual metal.

Ecological risk factor (E_r)	Risk grade	Reference
<40	Low potential ecological risk	(Mirzaei et al. 2014) [3]
40–80	Moderate potential ecological risk	
80–160	Considerable potential ecological risk	
160–320	High potential ecological risk	
≥320	Significantly high potential ecological risk	

Table S6 Grades of ecological risk index of heavy metal pollution.

Risk index (RI)	Risk grade	Reference
RI < 50	Low potential ecological risk	(Hakanson 1980) [4]
50 < RI ≤ 100	Moderate potential ecological risk	
100 < RI ≤ 200	High potential ecological risk	
RI > 200	Significantly high potential ecological risk	

Table S7 Distribution of species for leachates in the samples of Karachi city. Data calculated using PHREEQC and database MINTEQ. Values in molality.

Species	K.R (1)	K.R (2)	K.S (1)	K.S (2)	K.H (1)	K.H (2)	K.O (1)	K.O (2)
**As**								
**As (III)**								
H_3AsO_3	1.280E-03	9.310E-04	9.783E-04	1.853E-03	1.582E-04	2.416E-12	8.726E-04	1.340E-03
H_2AsO_3-	1.393E-07	1.408E-07	1.238E-07	5.962E-08	4.837E-08	2.014E-16	1.493E-07	1.373E-07
$H_4AsO_3^+$	8.775E-08	8.869E-08	7.795E-08	3.755E-08	3.046E-08	1.268E-16	9.406E-08	8.650E-08
$HAsO_3^{-2}$	5.543E-13	1.504E-12	7.735E-13	6.134E-15	4.266E-12	3.599E-22	2.312E-12	4.571E-13
**As (v)**								
$H_2AsO_4^-$	8.012E-02	8.167E-02	7.193E-02	3.475E-02	1.876E-01	1.163E-10	8.649E-02	7.989E-02
$HAsO_4^{-2}$	2.437E-02	6.671E-02	3.437E-02	2.734E-04	2.782E-02	1.590E-11	1.024E-01	2.033E-02
H_3AsO_4	7.937E-05	4.898E-05	5.688E-05	1.632E-04	6.857E-03	1.687E-13	4.257E-05	8.634E-05
AsO_4^{-3}	5.091eE-06	7.228E-05	1.524E-05	1.291E-10	5.081E-06	8.750E-16	2.057E-04	3.153E-06
**Pb**								
$PbOH^+$	3.917E-05	3.549E-06	5.938E-06	1.080E-05	4.927E-07	7.093E-13	2.626E-06	4.010E-05
$Pb(OH)_4^{-2}$	7.667E-22	1.914E-22	1.885E-22	5.692E-24	2.141E-22	6.318E-30	2.042E-22	6.793E-22
Pb^{+2}	1.831E-02	5.086E-04	1.660E-03	2.300E-02	2.434E-06	6.948E-10	2.265E-04	2.224E-02
$Pb_3(OH)_4^{+2}$	3.929E-09	4.097E-12	1.608E-11	2.466E-11	2.197E-14	1.794E-32	1.876E-12	4.016E-09
**Cr**								
**Cr (III)**								
Cr^{+3}	4.256E-01	1.805E-01	1.245E-01	3.819E-02	4.006E-01	9.182E-10	1.858E-01	9.393E+00
$Cr(OH)^{+2}$	4.057E-03	3.344E-04	5.649E-04	1.631E-01	2.183E-05	3.335E-11	1.853E-04	1.219E-01
$CrH_2PO_4^{+2}$	5.206E-04	6.177E-05	1.006E-04	9.065E-04	3.833E-06	9.660E-16	3.544E-05	8.336E-03
$Cr(OH)_3$	1.656E-10	3.721E-12	1.290E-11	8.950E-07	1.432E-14	3.991E-18	1.254E-12	6.461E-09
**Cr (VI)**								
$HCrO_4^-$	1.313E-31	4.134E-33	1.201E-32	2.127E-28	3.190E-35	2.434E-39	1.574E-33	4.881E-30
$CrO_3HPO_4^{-2}$	1.920E-32	2.337E-33	3.829E-33	3.482E-32	1.415E-34	000	1.335E-33	3.181E-31
CrO_4^{-2}	4.546E-34	4.426E-36	2.514E-35	3.402E-30	1.168E-39	000	1.013E-36	2.029E-32
H_2CrO_4	1.371E-36	3.105E-38	1.079E-37	7.508E-33	1.186E-40	000	1.045E-38	5.408E-35

Table S8 Distribution of species for leachates in the samples of Shikarpur city. Data calculated using PHREEQC and database MINTEQ. Values in molality.

Species	S.R (1)	S.R (2)	S.S (1)	S.S (2)	S.H (1)	S.H (2)	S.O (1)	S.O (2)
**As**								
**As (III)**								
H_3AsO_3	6.391E-04	4.045E-04	8.178E-05	6.300E-04	2.175E-05	5.577E-05	1.358E-04	5.328E-04
$H_2AsO_3^-$	7.016E-08	8.808E-08	1.266E-08	9.585E-08	8.888E-09	2.100E-08	4.261E-08	9.732E-08
$H_4AsO_3^+$	4.419E-08	5.547E-08	7.972E-09	6.037E-08	5.598E-09	1.322E-08	2.684E-08	6.129E-08
$HAsO_3^{-2}$	2.865E-13	2.806E-12	1.449E-13	1.042E-12	1.873E-12	3.458E-12	4.063E-12	1.831E-12
**As (v)**								
$H_2AsO_4^-$	4.057E-02	5.088E-02	7.257E-03	5.535E-02	5.097E-03	1.205E-02	2.451E-02	5.636E-02
$HAsO_4^{-2}$	1.267E-02	1.240E-01	6.351E-03	4.602E-02	8.213E-02	1.517E-01	1.787E-01	8.108E-02
H_3AsO_4	3.968E-05	1.682E-05	4.197E-06	3.288E-05	5.441E-07	1.501E-06	4.271E-06	2.496E-05
AsO_4^{-3}	2.765E-06	8.301E-04	7.719E-06	5.136E-05	1.282E-02	1.570E-02	7.436E-03	2.257E-04
**Pb**								
$PbOH^+$	2.500E-07	1.228E-06	1.689E-06	2.998E-08	1.407E-07	2.325E-07	4.603E-07	2.264E-06
$Pb(OH)_4^{-2}$	5.109E-24	1.951E-22	9.427E-23	1.624E-24	1.446E-22	1.872E-22	2.161E-22	2.141E-22
Pb^{+2}	1.130E-04	3.548E-05	2.233E-04	4.213E-06	1.250E-07	3.423E-07	1.965E-06	1.471E-04
$Pb_3(OH)_4^{+2}$	1.036E-15	2.430E-13	4.466E-13	2.472E-18	6.814E-16	2.837E-15	1.838E-14	1.283E-12
**Cr**								
**Cr (III)**								
Cr^{+3}	9.179E-04	3.263E-01	7.355E-04	2.904E-01	8.648E-01	6.371E-01	4.090E-01	1.995E-01
$Cr(OH)^{+2}$	8.422E-06	9.745E-05	1.201E-06	5.202E-04	1.100E-05	1.223E-05	1.957E-05	1.437E-04
$CrH_2PO_4^{+2}$	4.735E-07	1.820E-05	1.544E-07	4.478E-05	1.577E-06	1.899E-06	3.408E-06	2.772E-05
$Cr(OH)_3$	3.358E-13	2.506E-13	1.191E-14	5.605E-12	2.244E-15	3.471E-15	1.157E-14	7.494E-13
**Cr (VI)**								
$HCrO_4^-$	2.700E-34	3.993E-34	1.339E-35	6.238E-33	6.663E-36	9.499E-36	2.644E-35	1.004E-33
$CrO_3HPO_4^{-2}$	1.776E-35	6.808E-34	5.644E-36	1.672E-33	5.767E-35	6.958E-35	1.258E-34	1.044E-33
CrO_4^{-2}	9.094E-37	8.590E-38	1.308E-38	6.523E-36	000	1.034E-40	8.365E-40	4.869E-37
H_2CrO_4	2.794E-39	2.084E-39	000	4.657E-38	000	000	000	6.246E-39

References

1. Midander, K., et al., *Metal release from stainless steel particles in vitro—influence of particle size.* Journal of Environmental Monitoring, 2007. **9**(1): p. 74-81.
2. Rehman, A., et al., *Characterizing pollution indices and children health risk assessment of potentially toxic metal(oid)s in school dust of Lahore, Pakistan.* Ecotoxicology and Environmental Safety, 2020. **190**: p. 110059.
3. Mirzaei, R., et al., *Ecological risk of heavy metal hotspots in topsoils in the Province of Golestan, Iran.* Journal of Geochemical Exploration, 2014. **147**: p. 268-276.
4. Hakanson, L., *An ecological risk index for aquatic pollution control.a sedimentological approach.* Water Research, 1980. **14**(8): p. 975-1001.
